# Selection for Resistance to a Glyphosate-Containing Herbicide in Salmonella enterica Does Not Result in a Sustained Activation of the Tolerance Response or Increased Cross-Tolerance and Cross-Resistance to Clinically Important Antibiotics

**DOI:** 10.1128/AEM.01204-20

**Published:** 2020-11-24

**Authors:** Judith Pöppe, Katrin Bote, Abhinaya Ramesh, Jayaseelan Murugaiyan, Benno Kuropka, Michael Kühl, Paul Johnston, Uwe Roesler, Olga Makarova

**Affiliations:** a Institute of Animal Hygiene and Environmental Health, Centre for Infection Medicine, Freie Universität Berlin, Berlin, Germany; b Department of Biology & Biotechnology, SRM University-AP, Andhra Pradesh, India; c Institute for Chemistry and Biochemistry, Freie Universität Berlin, Berlin, Germany; d Evolutionary Biology, Institute for Biology, Freie Universität Berlin, Berlin, Germany; e Berlin Center for Genomics in Biodiversity Research, Berlin, Germany; f Leibniz-Institute of Freshwater Ecology and Inland Fisheries, Berlin, Germany; Rutgers, The State University of New Jersey

**Keywords:** glyphosate, *Enterobacteriaceae*, resistance, tolerance, fitness costs, experimental evolution

## Abstract

Evolution of bacterial tolerance to antimicrobials precedes evolution of resistance and may result in cross-tolerance, cross-resistance, or collateral sensitivity to other antibiotics. Transient exposure of gut bacteria to glyphosate, the world’s most widely used herbicide, has been linked to the activation of the stress response and changes in susceptibility to antibiotics. In this study, we investigated whether chronic exposure to a glyphosate-based herbicide (GBH) results in resistance, a constitutive activation of the tolerance and stress responses, and cross-tolerance or cross-resistance to antibiotics. Of the 10 farm animal-derived clinical isolates of Salmonella enterica subjected to experimental evolution in increasing concentrations of GBH, three isolates showed stable resistance with mutations associated with the glyphosate target gene *aroA* and no fitness costs. Global quantitative proteomics analysis demonstrated activation of the cellular tolerance and stress response during the transient exposure to GBH but not constitutively in the resistant mutants. Resistant mutants displayed no cross-resistance or cross-tolerance to antibiotics. These results suggest that while transient exposure to GBH triggers cellular tolerance response in Salmonella enterica, this response does not become genetically fixed after selection for resistance to GBH and does not result in increased cross-tolerance or cross-resistance to clinically important antibiotics under our experimental conditions.

**IMPORTANCE** Glyphosate-based herbicides (GBH) are among the world’s most popular, with traces commonly found in food, feed, and the environment. Such high ubiquity means that the herbicide may come into contact with various microorganisms, on which it acts as an antimicrobial, and it may select for resistance and cross-resistance to clinically important antibiotics. It is therefore important to estimate whether the widespread use of pesticides may be an underappreciated source of antibiotic-resistant microorganisms that may compromise efficiency of antibiotic treatments in humans and animals.

## Abstract (precis)

Glyphosate-based herbicides (GBH) are among the world’s most popular, with traces commonly found in food, feed, and the environment. Such high ubiquity means that the herbicide may come into contact with various microorganisms, on which it acts as an antimicrobial, and it may select for resistance and cross-resistance to clinically important antibiotics. It is therefore important to estimate whether the widespread use of pesticides may be an underappreciated source of antibiotic-resistant microorganisms that may compromise efficiency of antibiotic treatments in humans and animals.

## INTRODUCTION

Antimicrobial resistance (AMR) is a complex problem and a major existential threat as described by the World Economic Forum (https://www.weforum.org/reports/the-global-risks-report-2020). Chronic exposure of bacteria to sublethal concentrations of antimicrobials has long been identified as the major driver of the *de novo* evolution of resistance and cross-resistance to antibiotics (1). Tolerance is a transient phenotypic ability of the bacterial population to tolerate antimicrobials associated with general stress response (2). It has recently been shown that evolution of tolerance in response to subinhibitory concentrations of antibiotics precedes and facilitates emergence of resistance (3), making it an important but underappreciated contributor to AMR.

Glyphosate-based herbicides (GBH) are among the world’s most popular herbicides (4). While the potential toxic effects of glyphosate on humans, animals, and the environment are subjects of heated scientific and public debates (5, 6), its effects on gut bacteria have only recently attracted attention, despite its known antimicrobial properties (7) and frequent exposure through food and feed (8). Indeed, we recently found that Salmonella enterica sampled after the introduction of GBH into agricultural practice tended to have higher levels of resistance to glyphosate and GBH than the historical isolates from the preglyphosate era (9), while direct exposure to GBH has been shown to enrich for pathogenic bacteria in the gut (10) and change susceptibility to antibiotics in S. enterica and Escherichia coli through the activation of AcrAB efflux pumps (11), which are known to be involved in drug tolerance and resistance (12–14).

Intrigued by the findings of Kurenbach et al. (11), who found that transient exposure to subinhibitory concentrations of GBH resulted in altered antibiotic susceptibility profiles, and by the pervasive nature of glyphosate contamination, we sought to investigate whether chronic exposure to GBH results in the genetic fixation of this tolerance response and thereby may permanently compromise the efficiency of antibiotics.

## RESULTS AND DISCUSSION

**FIG 1:**
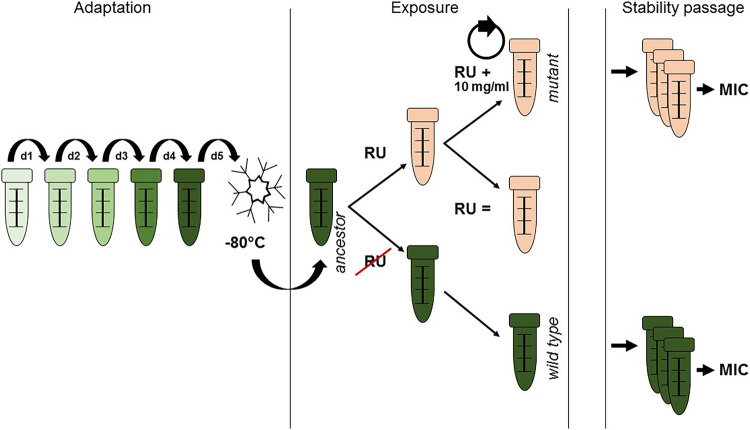
Schematic representation of the evolution experiment. Bacterial cultures were preadapted to the experimental conditions prior to the evolution experiment, where evolving populations were passaged daily 1:100 with the same Roundup LB Plus (RU) concentration and a concentration that was increased by 10 mg/ml, along with the nonselected controls. After the evolution experiments, populations were passaged in the absence of the herbicide and assessed by MIC testing for stability of resistance.

First, we attempted to obtain stable mutants resistant to GBH. For this, 10 clinical isolates of S. enterica from farm animals were passaged daily at increasing concentrations of the GBH Roundup LB Plus (RU), starting from 1/2× to 1/4× the MIC (20 mg/ml RU, equivalent to isopropylamine salt of glyphosate), depending on the strain, along with nonselected wild-type controls (Fig. 1). The overall dynamics of adaptation was slow and marked by early extinctions. Although all isolates were initially able to grow at 60 mg/ml, only three demonstrated a 2- to 4-fold MIC increase after the “stability of resistance” passage (in the absence of GBH) (Table 1). These data suggest that although evolution of resistance to GBH does not occur easily, it nonetheless has the potential to become fixed in resistant isolates.

**TABLE 1:** Dynamics of adaptation in the evolution experiment^*a*^

Serovar	Isolate no.	MIC before expt (mg/ml)	Highest concn with visible growth (mg/ml)	MIC after expt (mg/ml)	MIC after stability passage (mg/ml)	Day of extinction
***S*. Typhimurium**	**12468**	**40**	**90**	**160**	**160**	**17**
*S*. Typhimurium	12469	40	70			7
*S*. Typhimurium	12470	40	70			7
*S*. Typhimurium	12471	40	80			22
***S*. Typhimurium**	**12472**	**40**	**80**	**80**	**160**	**24**
*S*. Typhimurium	12473	40	60			5
*S*. Enteritidis	12538	80	80	80	80	10
***S*. Enteritidis**	**12539**	**80**	**70**	**80**	**160**	**10**
*S*. Enteritidis	12541	40	80			13
*S*. Enteritidis	12543	40	60			5

a The number of passages equals the number of days of the experiment before extinction. Resistant isolates that were subjected to whole-genome resequencing are in bold.

To gain insights into the molecular mechanisms of resistance, we sequenced the resulting GBH-resistant mutants and their respective ancestors. All three strains had missense mutations either upstream of (S. enterica serovar Typhimurium 12468M and S. enterica serovar Enteritidis 12539M) or inside (*S*. Typhimurium 12472M) *aroA*, the gene encoding the molecular target of glyphosate (15, 16). Additionally, mutations in the genes frequently associated with stress response and tolerance (17, 18) were also found (Table 2; Table S1): truncation of *rpoS* in *S*. Typhimurium 12472M, which encodes RNA polymerase sigma factor, a master regulator of the general stress response (19), and a missense mutation in *rcsB* in *S*. Typhimurium 12468M, encoding the transcriptional regulator of a two-component system. These data suggest that while evolution of resistance to GBH converges at the target gene and functional levels (various genes related to stress and tolerance response), individual strains employ different strategies to achieve this adaptation.

**TABLE 2:** Overview of the nonsynonymous mutations detected in GBH-resistant mutants^*a*^

Isolate	Affected gene	Type and location of mutations in coding and protein sequences	Protein function
*S*. Typhimurium 12468M	*aroA*	SNP in scaffold_16:G41393A upstream of *aroA*	3-Phosphoshikimate 1-carboxyvinyltransferase
	*rcsB*	Missense variant c.530G>T/651 p.Arg177Leu/216	Two-component system transcriptional regulator RcsB
*S*. Typhimurium 12472M	*rpoS*	Stop gained c.361G>T/993 p.Glu121*/330	RNA polymerase sigma factor RpoS
	*aroA*	Missense variant c.289A>G/1284 p.Thr97Ala/427	3-Phosphoshikimate 1-carboxyvinyltransferase
	Multiple	91 missense mutations in prophage genes	Prophage genes
*S*. Enteritidis 12539M	*aroA*	SNP in scaffold_0:G272493A upstream of *aroA*	3-Phosphoshikimate 1-carboxyvinyltransferase

a Stable resistant mutants (single colonies after the stability passage) were subjected to whole-genome resequencing and compared to the genomes of the nonevolved controls and ancestors. Mutation locations are indicated as follows: type of mutation, followed by the variant of nucleotide or amino acid present in the ancestor, position (relative to the gene or protein start) at which the substitution occurred, substituted nucleotide or amino acid in the mutant relative to the complete nucleotide coding (c) and translated protein (p) sequences (after the slash). Mutations in the *aroA* gene encoding the molecular target of glyphosate are shaded in gray. Note that *S*. Typhimurium 12468 and *S*. Enteritidis 12539 appear to have the same mutation in the intergenic space upstream of *aroA*, while *S*. Typhimurium 12472 has the amino acid substitution in the location previously associated with resistance to glyphosate (47), suggesting a high degree of convergent evolution between these environmental isolates. SNP, single-nucleotide polymorphism.

**FIG 2:**
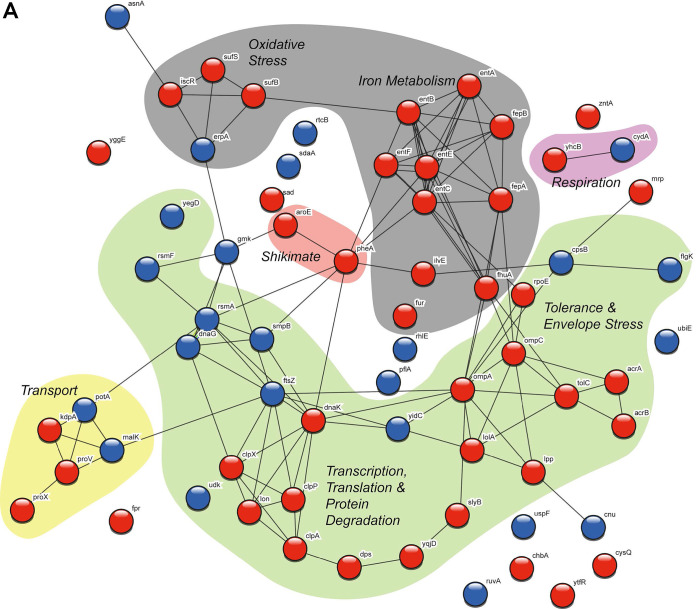

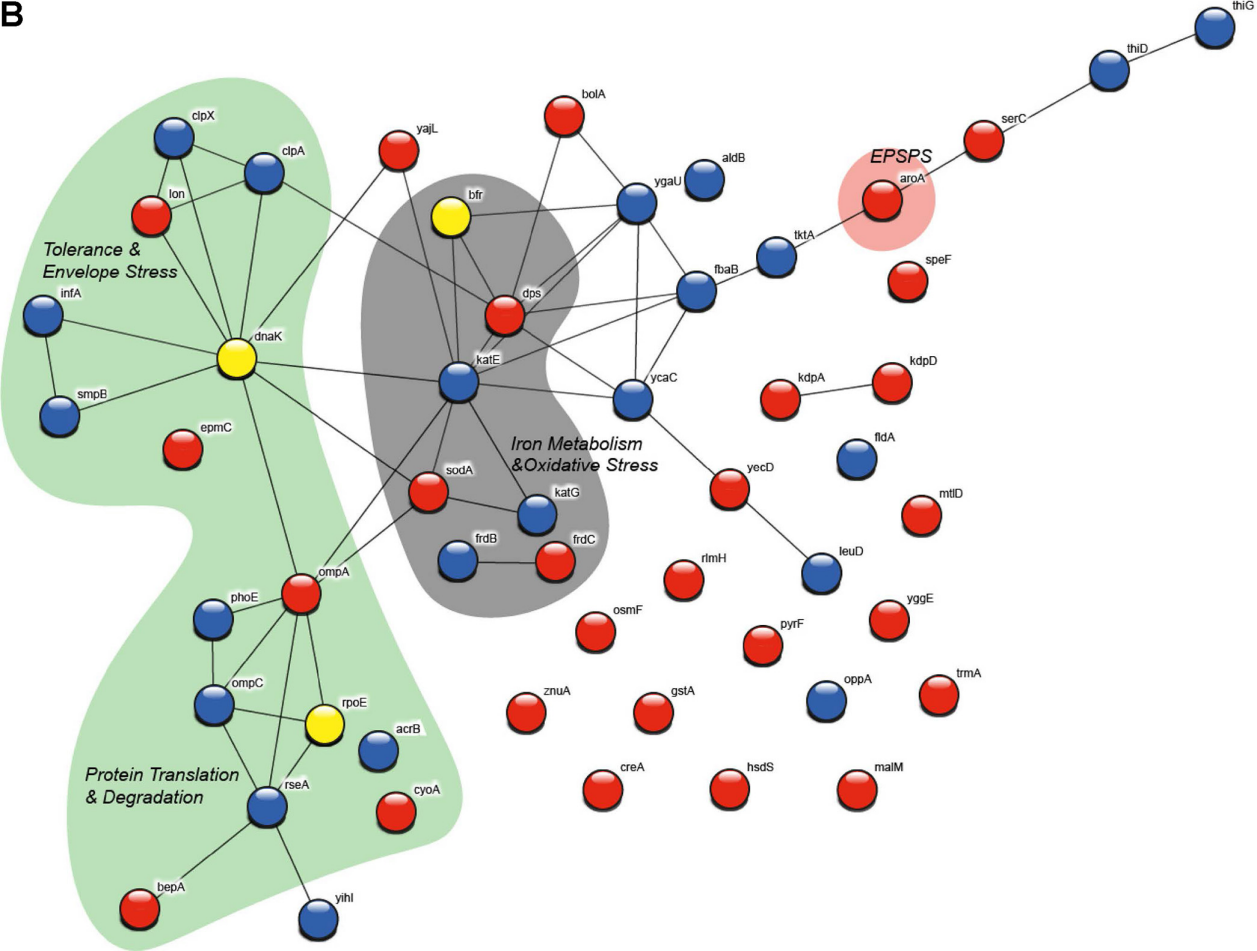
STRING network analysis of the proteome. The combined proteome of four challenged ancestors (A) and that of the three resulting constitutively GBH-resistant mutants (B) are shown, representing proteins involved in the processes known to be affected by glyphosate (production of aromatic amino acids, chelation of iron, and stress response in bacteria) and the 10 most up- and downregulated proteins for each strain. Blue spheres represent downregulated proteins, red spheres represent upregulated proteins, and yellow spheres are proteins which are upregulated in one strain and downregulated in another strain. Proteins highlighted with the same color belong to a functional group.

To probe more deeply into what cellular processes are affected by resistance to GBH, we performed global label-free quantitative proteomics analysis of the resistant mutants and their ancestors in the presence and absence of subinhibitory (1/4× MIC) concentrations of GBH (Table S2; Fig. S1 and S2). We wondered whether the acute response to the sublethal concentration of GBH in the sensitive ancestor simply became amplified and constitutively fixed in resistant mutants following chronic exposure. While upregulation of proteins involved in oxidative stress response was consistent in both challenged sensitive ancestors and constitutive GBH-resistant mutants, the overall cellular responses were vastly different (Fig. 2A and B), suggesting that evolution of resistance to GBH does not simply result in fixation of the acute response to GBH stress.

When we searched the combined proteome of the four challenged ancestors (three ancestors of the resistant mutants—12468A, 12472A, and 12539A—and one ancestor of the extinct line 12538A used as a control) for proteins involved in the processes known to be affected by glyphosate (production of aromatic amino acids, chelation of iron, and stress response in bacteria) and plotted them together with the 10 most up- and downregulated proteins for each strain using STRING network analysis (20), we found a striking convergence at the tolerance response (Fig. 2A). The acriflavine resistance AcrAB multidrug efflux pump, which is associated with tolerant persister state in nongrowing and nondividing cells, including herbicide paraquat-induced tolerance (21, 22), was upregulated in all challenged ancestral isolates. This is consistent with the findings of Kurenbach et al., who demonstrated that exposure of *Enterobacteriaceae* to subinhibitory concentrations of GBH resulted in activation of efflux pumps and was associated with increased antibiotic tolerance (11), while deletion of *acrA*, *acrB*, and *tolC* (but not of *ompF* and *acrD*) caused a reduction in the MIC of GBH (23). Furthermore, TolC, which is frequently associated with tolerance, was also upregulated in all but one strain. Among other upregulated proteins were those involved in uptake and metabolism of iron and other divalent trace metals (siderophores and a number of transporters), consistent with the chelating effects of glyphosate (24). General envelope (RpoE, OmpA, LolA, Lpp, and SlyB), osmotic (osmolarity response proteins and osmoprotectants YehZ and OsmY), and oxidative (SufB, SufC, and SufS) stress response proteins were also upregulated, as well as respiration (CydAB), DNA recombination (RuvAB), and cell division (FtsZ) proteins. No effects directly on the target of glyphosate (3-phosphoshikimate1-carboxyvinyltransferase or 5-enolpyruvylshikimate-3-phosphate synthase [EPSPS]) were found in any of the strains, although other proteins involved in the shikimate pathway (chorismate mutase and synthase) were upregulated in all strains except *S*. Typhimurium 12468.

Recently, a relationship between stress response, iron limitation, and amino acid uptake was demonstrated (25). Banerjee et al. reported that for pathogenic E. coli, survival in the urinary tract is linked to the stress response-mediated ability to increase amino acid uptake under iron-limiting conditions (25). It is conceivable that similar processes occur in *Salmonella* exposed to glyphosate, which acts as a potent iron chelator, and these would be consistent with the increased stress response and iron metabolism observed in our experiments. In short, our data strongly suggest that activation of iron limitation and tolerance response precedes activation of expression of the specific glyphosate target following acute GBH stress in a rich medium.

In contrast, similar analysis of the combined proteomes of the three constitutively GBH-resistant mutants (*S*. Enteritidis 12539M, *S*. Typhimurium 12468M, and *S*. Typhimurium 12472M) and their ancestors revealed few similarities between the isolates, with the exception of the molecular target of glyphosate EPSPS, which was upregulated in all three mutants (Fig. 2B; Table S2; Fig. S2). Interestingly, iron metabolism-related proteins were not as strongly affected by the evolution of resistance to GBH, despite a strong activation of these proteins in the challenged sensitive ancestors. Nonetheless, bacterioferritin, which is used for storage of intracellular iron, was upregulated in resistant *S*. Typhimurium 12468M but downregulated in resistant *S*. Typhimurium 12472M. It is important to note that none of the ferritins were upregulated during the GBH exposure of the sensitive ancestors, suggesting that *Salmonella*’s short-term response to iron limitation by glyphosate chelation is to increase transport but not storage of iron. There were fewer proteins involved in tolerance and envelope stress represented in this data set, and those that were present tended to be downregulated, in contrast to the response in challenged ancestors. Altogether, proteomics data suggest that chronic exposure to GBH results in constitutive fixation of the resistance traits associated with the direct effects of the herbicide on bacteria (EPSPS and iron chelation) but not the tolerance response, which is activated by the presence of GBH in both sensitive ancestors and resistant mutants.

It was demonstrated previously that evolution of tolerance precedes evolution of resistance (3) and may result in cross-tolerance (26) and collateral sensitivity (27) to other antimicrobials. Indeed, our experimental evolution resulted in resistance to GBH, while proteomics demonstrated activation of the tolerance response upon transient exposure to GBH in both ancestors and mutants. To check whether genetically fixed resistance to GBH affects cross-tolerance and cross-resistance/collateral sensitivity, we subjected the ancestors and the mutants to TDtest assays (28) and MIC testing by Vitek automated susceptibility testing (AST) (in the absence of GBH) against a number of antibiotics relevant to human medicine. We found no tolerant bacteria in TDtest assays with the β-lactam antibiotics ceftazidime (CAZ; third-generation cephalosporin) and cefepime (FEP; 4th-generation cephalosporin) or with rifampin (RIF) or colistin (CT; also known as polymyxin) (Fig. S3), consistent with the lack of tolerance response at the proteome level. Interestingly, we found colonies in tolerance assays with fosfomycin (FOS) which upon retesting by MIC testing displayed an elevated level of resistance and are likely spontaneous mutants (Fig. S4), a phenomenon frequently described for this antibiotic (29). MIC assays by Vitek showed no changes between nonchallenged ancestors and mutants with the exception of the isolate 12472, where the mutant had a decreased MIC of piperacillin (Table 3), likely a sign of collateral sensitivity. In short, GBH resistance had no effect on cross-tolerance and cross-resistance to antibiotics.

**TABLE 3:** Susceptibilities to antibiotics in GBH mutants and ancestors

Agent	Antibiotic class	MIC (mg/liter) and category for^*a*^:					
		*S*. Typhimurium 12468		*S*. Typhimurium 12472		*S*. Enteritidis 12539	
		Ancestor (40)	Mutant (160)	Ancestor (40)	Mutant (160)	Ancestor (80)	Mutant (160)
Piperacillin	β-Lactam	≥128 R	≥128 R	≥128 R	≥128 R	≤4 S	≤4 S
Piperacillin-tazobactam	β-Lactam–β-lactamase-inhibitor	≤4 S	≤4 S	**8 S**	**≤4 S**	≤4 S	≤4 S
Cefotaxime	3rd-generation cephalosporin	≤1 S	≤1 S	≤1 S	≤1 S	≤1 S	≤1 S
Ceftazidime	3rd-generation cephalosporin	≤1 S	≤1 S	≤1 S	≤1 S	≤1 S	≤1 S
Cefepime	4th-generation cephalosporin	≤1 S	≤1 S	≤1 S	≤1 S	≤1 S	≤1 S
Aztreonam	Monobactam	≤1 S	≤1 S	≤1 S	≤1 S	≤1 S	≤1 S
Imipenem	Carbapenem	≤0.25 S	≤0.25 S	≤0.25 S	≤0.25 S	≤0.25 S	≤0.25 S
Meropenem	Carbapenem	≤0.25 S	≤0.25 S	≤0.25 S	≤0.25 S	≤0.25 S	≤0.25 S
Amikacin	Aminoglycoside	≤2 R	≤2 R	≤2 R	≤2 R	≤2 R	≤2 R
Gentamicin	Aminoglycoside	≤1 R	≤1 R	≤1 R	≤1 R	≤1 R	≤1 R
Tobramycin	Aminoglycoside	≤1 R	≤1 R	≤1 R	≤1 R	≤1 R	≤1 R
Ciprofloxacin	Fluoroquinolone	≤0.25 R	≤0.25 R	≤0.25 R	≤0.25 R	≤0.25 R	≤0.25 R
Tigecycline	Glycylcycline	≤0.5 S	≤0.5 S	≤0.5 S	≤0.5 S	≤0.5 S	≤0.5 S
Fosfomycin	Epoxide	≤16 S	≤16 S	≤16 S	≤16 S	≤16 S	≤16 S
Trimethoprim-sulfamethoxazole	Folate inhibitor-sulfonamide	≤20 S	≤20 S	≤20 S	≤20 S	≤20 S	≤20 S

a MICs were determined using the Vitek2 AST N-248 panel of antibiotics. Changes in MIC between ancestors and mutants are in bold. Numbers in parentheses are concentrations of Roundup LB Plus, in milligrams per milliliter. R, resistant; S, susceptible.

**FIG 3:**
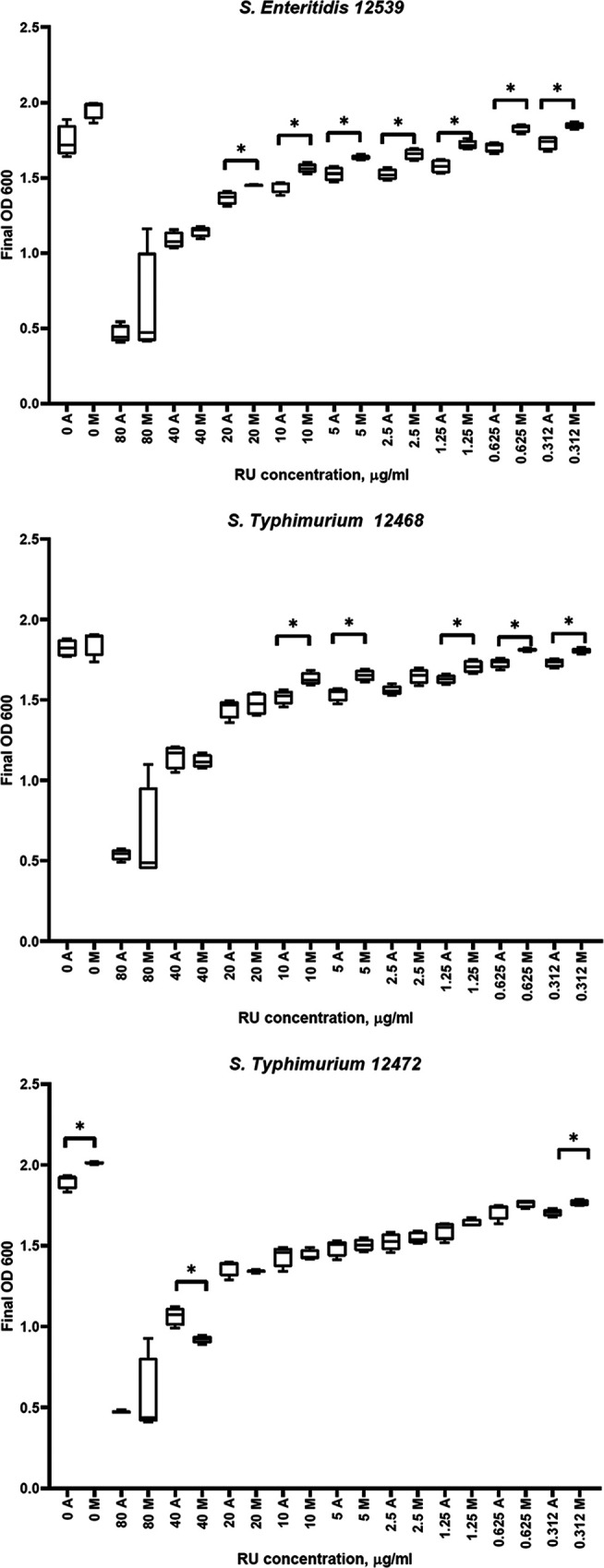
Fitness costs. Final biomass (OD_600_ values at 16 h) was used as a proxy for bacterial fitness in the presence and absence of Roundup LB Plus (RU). M, mutant; A, ancestor. Asterisks indicate statistical significance as determined by the *t* test (*P* < 0.05). Note the absence of fitness costs in the absence of RU and the subtle fitness advantage at certain subinhibitory concentrations of RU.

Evolution of resistance is often accompanied by fitness costs, meaning that resistant mutants would not survive as well as their sensitive ancestors. While subinhibitory concentrations of antibiotics are usually necessary for AMR selection, they are not always needed for maintenance, once fitness costs become sufficiently reduced by compensatory mechanisms (30). To test whether resistance to GBH is associated with fitness costs or advantages, we compared the growth of resistant mutants and their GBH-sensitive ancestors in the absence and presence of several concentrations of the selective agent. For this, we performed growth curves of ancestors and mutants individually and used the final biomass at 16 h as a proxy for fitness. In the absence of GBH, two strains (*S*. Enteritidis 12539M and *S*. Typhimurium 12468M) demonstrated no fitness costs, and *S*. Typhimurium 12472M displayed a fitness advantage. All three resistant mutants had a higher biomass than the ancestors at 80 mg/ml GBH (the highest level of resistance achieved in the experiment and the highest concentration tested), as well as small fitness advantages across the range of subinhibitory concentrations (0.312 to 20 mg/ml) (Fig. 3). Additionally, we performed a growth rate inhibition analysis of the growth curves in the presence and absence of GBH (Fig. S5). This method is independent of cell division rate and assay duration and is considered more robust than traditional 50% inhibitory concentration (IC_50_) determination for estimation of cellular responses to drugs (31, 32). Similarly to the biomass analyses, comparison of the growth rate inhibition of ancestral and mutant strains in the presence of nine different concentrations of GBH showed no statistically significant differences. Our data indicate that stable resistance to GBH not only is possible but also is free of fitness costs or even advantageous, in both the presence and absence of GBH. While this suggests that the resistant GBH mutants may persist in the environment even when the selective pressure is not present, a competition assay between the mutant and the ancestor *in vivo* would provide evidence as to whether this is indeed the case, and this is the subject of further investigations.

Our study demonstrated that transient exposure of pathogenic Salmonella enterica bacteria to subinhibitory concentrations of the herbicide GBH in a rich growth medium and at a physiological (neutral) pH (a situation resembling *in vivo* conditions) readily elicits a tolerance response at the cellular level and upregulation of the AcrAB-TolC efflux system, while chronic exposure to GBH results in selection for GBH resistance but not cross-tolerance or cross-resistance to clinically relevant antibiotics. Importantly, although our results indicate that GBH resistance does not occur easily and is relatively low level (2 to 4× MIC increase), it is stable and is associated with no fitness costs in the absence of GBH and even a fitness advantage in its presence.

Our results are in line with the findings of Randall et al. (33), who demonstrated that exposure of Salmonella enterica serovar Typhimurium to commonly used farm disinfectants resulted in upregulation of the AcrAB-TolC efflux system, while selection for resistance to these biocides largely did not result in resistance to multiple antibiotics. Similar to our results, these biocide-resistant mutants did not show any fitness losses relative to parent strains. Kurenbach et al. demonstrated through the use of efflux pump and stress response regulator reporter assays and efflux pump gene deletion experiments that efflux plays an important role in the response of *Enterobacteriaceae* to GBH (11, 23), which is also in agreement with our data showing upregulation of the AcrAB-TolC efflux pump.

Interestingly, no noticeable upregulation of efflux pump genes was found in a transcriptomic study of E. coli in the presence of glyphosate (34), where largely energy- and metabolism-related genes were downregulated, while cell motility and chemotaxis-related genes were upregulated. This discrepancy may be explained by the fact that different types and concentrations of GBH were used: 200 mM glyphosate (or 33.814 mg/ml) (34) versus 1,250 ppm (or 1.25 mg/ml) complete formulation Roundup weed killer (11) and 10 to 20 mg/ml Roundup LB Plus in the present study, both containing 360 g/liter isopropylamine salt. Indeed, it has been demonstrated that inert wetting agents found in complete formulations may also change bacterial sensitivity to antibiotics (23), highlighting the importance of making a distinction between the effects of the active ingredient and the complete formulation. Additionally, differences in the sensitivity of the methods, culturing conditions, and bacterial strains may also offer an explanation. At the same time, very few changes were found in the transcriptome of E. coli during the heterologous expression of the resistant *aroA* variant in the absence of the herbicide (35). This mirrors our observations of the proteomes in the resistant mutants in the absence of GBH, where only the proteins directly involved in resistance to glyphosate were differentially expressed.

Overall, there appears to be a consensus that while acute exposure to GBH triggers activation of efflux pumps and stringent response, no such effects are observed during the constitutive expression of the resistant EPSPS variant in the absence of GBH. It is also noteworthy that although the GBH concentrations used in this study exceed the concentrations typically found in animal feed (8), they are not unlikely and can be found during handling of the undiluted herbicide. Interestingly, it has been reported that exposure to higher GBH concentrations decreases the genome-wide mutation rate in E. coli, suggesting that long-term exposure to GBH does not compromise bacterial genome stability (36). This is in agreement with our observations of slow resistance evolution at increasingly higher concentrations of GBH, frequent extinctions, few observed mutations, and no cross-resistance to antibiotics, assuming that increased mutation supply is a prerequisite for evolution, including evolution of antibiotic resistance (37). Nonetheless, while this study provides important hints for extended risk assessment of ubiquitous herbicides such as glyphosate, the findings may be limited to the specific experimental conditions, and therefore, more studies with a broader range of bacterial species are needed to determine the relevance of these findings *in vivo*.

## MATERIALS AND METHODS

### Bacterial strains and culturing conditions.

Ten isolates of S. enterica serovars Enteritidis and Typhimurium originally isolated from pig feces were provided by the German Federal Institute for Risk Assessment. All cultures were grown in Mueller-Hinton I (MHI) medium (CM0405; Oxoid GmbH, Wesel, Germany) and incubated at 37°C with moderate shaking, unless stated otherwise.

### Experimental evolution.

Single colonies were isolated from blood agar plates and passaged daily 1:100 in 5 ml MHI in a 50-ml Falcon tube for 3 days to help bacteria adapt to the experimental conditions (referred to as the preadaptation passage). Roundup LB Plus (German license 024142-00; Monsanto) was used for experiments. After the preadaptation phase, the medium was supplemented with increasing concentrations of Roundup LB Plus, adjusted with NaOH to pH 7. The starting subinhibitory concentration for the challenge was 20 mg/ml glyphosate isopropylamine salt in Roundup LB Plus. Each day, 50 μl of the overnight culture was transferred into two new tubes, one with the same concentration of Roundup at which the visible growth occurred in the last passage and one with a concentration of glyphosate 10 mg/ml higher than that. Nonevolving controls were handled similarly with the exception that Roundup was absent. The experiment ended when no growth was visible after 24 h in both tubes. Roundup-selected isolates were then passaged 1:100 in the absence of the herbicide (referred to as the stability passage), followed by the determination of the MICs (Fig. 1).

### DNA preparation and whole-genome resequencing.

Isolates which remained Roundup resistant after the stability passage were subjected to whole-genome resequencing along with nonselected controls and ancestors (each as a single colony and a population) (Table S1). For colony sequencing, individual colonies were isolated from Mueller-Hinton agar plates before culturing in liquid medium. For population sequencing, a loopful of the frozen stock was directly cultivated in 3 ml MHI. DNA was extracted with a GeneMatrix bacterial and yeast genomic DNA purification kit (EURx Molecular Biology Products, Gdansk, Poland). DNA concentration was determined using Nanodrop at 260/280 nm. DNA integrity was ensured by gel electrophoresis (1% agarose). Isolated DNA was stored at −20°C until sequencing.

Sequencing libraries were constructed from 2 μg total genomic DNA using a TruSeq DNA PCR-free kit (Illumina) and sequenced for 600 cycles using a MiSeq at the Berlin Center for Genomics in Biodiversity Research.

Reference genomes were constructed by assembling sequencing reads using the a5-miseq pipeline (38) and annotated using prokka (39). The variant calling pipeline Snippy was used to identify mutations in the selection lines. Snippy uses bwa (40) to align reads to the reference genome and identifies variants in the resulting alignments using FreeBayes (41). Variants were verified using the breseq computational pipeline (42).

### Label-free quantitative proteomics analysis.

Isolates from ancestral and mutant lines frozen stocks were grown overnight in MHI at 37°C with shaking, diluted 1:100, and subcultured until an optical density of 0.5 was achieved. Each mutant-ancestor pair was allowed to grow for 25 min more in the presence and absence of Roundup LB Plus; each condition consisted of six biological replicates. Treatment samples were challenged with 1/4× MIC Roundup and had their pH adjusted with 5 M NaOH to neutral.

Following incubation, subcultures were centrifuged for 5 min at room temperature at maximum speed, and the supernatant was discarded. The pellet was washed with 1 ml PBS, followed by addition of 300 μl distilled water and 900 μl ethanol (EtOH) (100%). After 1 h of incubation at room temperature, samples were centrifuged for 10 min at room temperature at 10,000 × *g*. The supernatant was discarded, and the pellet was air dried and stored at −20°C until protein extraction.

Protein extraction was carried out with the ethanol-fixed cells. In brief, the cells were reconstituted with 100 μl each of acetonitrile (100%) and formic acid (75% [vol/vol]). The samples were sonicated on ice for 1 min (duty cycle, 1.0; amplitude, 100%) (UP100H; Hielscher Ultrasound Technology, Teltow, Germany) and centrifuged at 11,290 × *g* for 5 min at 4°C. The clear supernatant was collected, and the protein content was quantified using the Qubit method (Thermo Fisher Scientific, Germany) following the manufacturer’s recommendations.

In-solution trypsin digestion was carried out at room temperature as described elsewhere (43). The resultant trypsin-digested peptide products were first desalted by solid-phase extraction using C_18_ Empore disc cartridges (Supelco/Sigma-Aldrich, Taufkirchen, Germany) and dried under vacuum. Peptides were reconstituted in 10 μl of 0.05% trifluoroacetic acid (TFA)–2% acetonitrile, and 2 μl was analyzed by a reversed-phase nanoscale liquid chromatography system (Ultimate 3000; Thermo Scientific) connected to an Orbitrap Velos mass spectrometer (Thermo Scientific). Samples were injected and concentrated on a trap column (PepMap100 C_18_ [Thermo Scientific]; 3 μm, 100 Å, 75 μm [inside diameter], 2-cm length) equilibrated with 0.05% TFA–2% acetonitrile in water. After switching the trap column inline, liquid chromatography (LC) separations were performed on a capillary column (Acclaim PepMap100 C_18_ [Thermo Scientific]; 2 μm, 100 Å, 75 μm [inside diameter], 25-cm length) at an eluent flow rate of 300 nl/min. Mobile phase A contained 0.1% formic acid in water, and mobile phase B contained 0.1% formic acid in acetonitrile. The column was pre-equilibrated with 3% mobile phase B followed by an increase from 3 to 50% mobile phase B in 80 min. Mass spectra were acquired in a data-dependent mode using a single mass spectrometry (MS) survey scan (*m/z* 350 to 1,500) with a resolution of 60,000 in the Orbitrap and MS/MS scans of the 20 most intense precursor ions in the linear trap quadrupole. The dynamic exclusion time was set to 60 s, and automatic gain control was set to 1 × 10^6^ and 5,000 for Orbitrap-MS and LTQ-MS/MS scans, respectively. The acquired raw data files from mass spectrometry were processed using the MaxQuant-Andromeda software suite (version 1.6.0.16; Max Planck Institute of Biochemistry, Martinsried, Germany) (44).

Protein identification was carried out by searching MS and MS/MS data against FASTA files of protein sequences produced by translating annotated DNA sequences. The parametric settings were set for protein identification, as follows: mass tolerance, 7 ppm; MS and MS/MS ion tolerance, 0.5 Da; enzymes LysC and trypsin, both with two missed cleavage sites allowed for the database search; variable modification including oxidation of methionine and protein N-terminal acetylation; fixed modification including carbamidomethylation; target-decoy-based false discovery rate (FDR) for peptide and protein identification of 1% for peptides and proteins; and minimum peptide length, 7 amino acids. The software Perseus (version 1.6.1.1; Max Planck Institute of Biochemistry, Martinsried, Germany) (45) was used for identification of differentially expressed proteins. The MaxQuant result file (protein groups.txt) was imported into the Perseus software, and a reduction matrix was applied to remove proteins identified only by site and reverse and potential contamination. The intensity values were transformed to log_2_ values, and a reduction matrix based on signal detection in three of six replicates in any one of the group was applied. The two-way Student *t* test, error correction (*P* < 0.05), and FDR correction of the alpha error were applied through the Benjamini-Hochberg procedure for identification of differentially expressed proteins among the compared groups. The STRING online tool (v.11; https://string-db.org/) (20) was used to visualize proteins affected by Roundup in protein networks. For this, significantly differentially expressed proteins from the processes known to be affected by glyphosate and the 10 most up- and downregulated proteins for each strain were used.

### Antimicrobial susceptibility testing.

The MIC of Roundup LB Plus (German license 024142-00; Monsanto) was determined by the broth microdilution method described in reference 9 in MHI. Because GBH acidifies medium, the pH was adjusted to neutral with NaOH. The concentration of glyphosate was calculated based on the concentration of glyphosate isopropylamine salt in the herbicide formulation. Cross-resistance to a panel of antibiotics relevant to human medicine was determined via antibiotic susceptibility testing with the Vitek system (bioMérieux Deutschland GmbH, Nürtingen, Germany) using the test card Vitek 2 AST N-248. The tested antimicrobials were piperacillin, piperacillin-tazobactam, cefotaxime, ceftazidime, cefepime, aztreonam, imipenem, meropenem, amikacin, gentamicin, tobramycin, ciprofloxacin, moxifloxacin, tigecycline, fosfomycin, and trimethoprim-sulfamethoxazole.

### Tolerance detection test.

Tolerant colonies were detected by the tolerance detection (TD) test based on the semiquantitative method described in reference 28. The overnight culture for each isolate was prepared with three biological replicates, each of which was individually inoculated from a single cryopreserved stock. An appropriate quantity of cells was resuspended in 0.85% NaCl, and the optical density was adjusted to 0.5. The bacterial suspension was plated on dried Mueller-Hinton I agar plates containing 0.5% glucose. Ready-to-use discs for CAZ (30 μg per disc), FEP (30 μg per disc), and CT (10 μg per disc) were purchased from Oxoid (Thermo Fisher Scientific, Germany). Fosfomycin (200 μg per disc) and rifampin (10 μg per disc) diffusion discs were prepared by soaking blank discs with 20 μl of antibiotic stock solution per disc. A disc diffusion assay was performed by placing the discs on the lawn of bacteria and incubating them overnight at 37°C. After the overnight incubation, the antibiotic discs were replaced by discs containing 40% glucose and incubated overnight. The isolated colonies found in the zone of inhibition after the incubation with glucose discs represent the tolerant colonies.

### Fitness costs.

Growth curves were performed in a plate reader (Synergy HTX; BioTech Instruments, Germany). Ancestral and mutant lines were grown in tubes with 3 ml of MHI overnight with shaking. Subsequently, the overnight cultures were diluted 1:100 in MHI and grown for approximately 2 h until an optical density at 600 nm (OD_600_) of 0.5 was reached. An aliquot of 100 μl was transferred into the wells of a 96-well plate containing 100 μl MHI. Measurements were taken at 10-min intervals after a short period of shaking and incubation at 37°C for 16 h in the plate reader. The assays were performed in the absence and presence of Roundup LB Plus (2-fold dilutions from 0.3125 to 80 mg/ml). Four biological and technical replicates were used for each sample-treatment combination. As a proxy for fitness, mean values of the final biomass (OD_600_ at 16 h) were used. A *t* test was used to calculate statistical significance (*P* < 0.05) of the difference between ancestors and mutants using GraphPad Prism 8. Growth rate (GR) inhibition analysis was performed using GRcalculator (31). For this, three time points (2, 9, and 16 h) and 9 concentrations (0.3125, 0.625, 1.25, 2.5, 5.0, 10.0, 20.0, 40.0, and 80.0 mg/ml glyphosate in RU LB Plus) were used to calculate GR_50_, GR_max_, and GR_inf_ values (Fig. S5).

### Data availability.

Sequence data are available from the NCBI SRA under BioProject accession no. PRJNA485244. The mass spectrometry proteomics data have been deposited in the ProteomeXchange Consortium via the PRIDE (46) partner repository with the data set identifier PXD019463.

## Supplementary Material

Supplemental file 1

Supplemental file 2

## References

[1] Andersson DI, Hughes D 2010 Antibiotic resistance and its cost: is it possible to reverse resistance? Nat Rev Microbiol 8:260–271. doi:10.1038/nrmicro2319. 20208551

[2] Windels EM, Michiels JE, Van den Bergh B, Fauvart M, Michiels J 2019 Antibiotics: combatting tolerance to stop resistance. mBio 10:e02095-19. doi:10.1128/mBio.02095-19. 31506315 PMC6737247

[3] Levin-Reisman I, Ronin I, Gefen O, Braniss I, Shoresh N, Balaban NQ 2017 Antibiotic tolerance facilitates the evolution of resistance. Science 355:826–830. doi:10.1126/science.aaj2191. 28183996

[4] Benbrook CM 2016 Trends in glyphosate herbicide use in the United States and globally. Environ Sci Eur 28:3. doi:10.1186/s12302-016-0070-0. 27752438 PMC5044953

[5] Myers JP, Antoniou MN, Blumberg B, Carroll L, Colborn T, Everett LG, Hansen M, Landrigan PJ, Lanphear BP, Mesnage R, Vandenberg LN, Vom Saal FS, Welshons WV, Benbrook CM 2016 Concerns over use of glyphosate-based herbicides and risks associated with exposures: a consensus statement. Environ Health 15:19. doi:10.1186/s12940-016-0117-0. 26883814 PMC4756530

[6] Van Bruggen AHC, He MM, Shin K, Mai V, Jeong KC, Finckh MR, Morris JG 2018 Environmental and health effects of the herbicide glyphosate. Sci Total Environ 616–617:255–268. doi:10.1016/j.scitotenv.2017.10.309. 29117584

[7] Coggins JR, Abell C, Evans LB, Frederickson M, Robinson DA, Roszak AW, Lapthorn AP 2003 Experiences with the shikimate-pathway enzymes as targets for rational drug design. Biochem Soc Trans 31:548–552. doi:10.1042/bst0310548. 12773154

[8] EFSA. 2018 Evaluation of the impact of glyphosate and its residues in feed on animal health. EFSA J 16:e05283. doi:10.2903/j.efsa.2018.5283. 32625919 PMC7009421

[9] Pöppe J, Bote K, Merle R, Makarova O, Roesler U 2019 Minimum inhibitory concentration of glyphosate and a glyphosate-containing herbicide in salmonella enterica isolates originating from different time periods, hosts, and serovars. Eur J Microbiol Immunol 9:35–41. doi:10.1556/1886.2019.00005. PMC6563685 31223494

[10] Shehata AA, Schrödl W, Aldin AA, Hafez HM, Krüger M 2013 The effect of glyphosate on potential pathogens and beneficial members of poultry microbiota in vitro. Curr Microbiol 66:350–358. doi:10.1007/s00284-012-0277-2. 23224412

[11] Kurenbach B, Marjoshi D, Amábile-Cuevas CF, Ferguson GC, Godsoe W, Gibson P, Heinemann JA 2015 Sublethal exposure to commercial formulations of the herbicides changes in antibiotic susceptibility in Escherichia coli and Salmonella enterica serovar Typhimurium. mBio 6:e00009-15. doi:10.1128/mBio.00009-15. 25805724 PMC4453521

[12] Nolivos S, Cayron J, Dedieu A, Page A, Delolme F, Lesterlin C 2019 Role of AcrAB-TolC multidrug efflux pump in drug-resistance acquisition by plasmid transfer. Science 364:778–782. doi:10.1126/science.aav6390. 31123134

[13] Cohen NR, Lobritz MA, Collins JJ 2013 Microbial persistence and the road to drug resistance. Cell Host Microbe 13:632–642. doi:10.1016/j.chom.2013.05.009. 23768488 PMC3695397

[14] Fernandes P, Ferreira BS, Cabral JMS 2003 Solvent tolerance in bacteria: role of efflux pumps and cross-resistance with antibiotics. Int J Antimicrob Agents 22:211–216. doi:10.1016/S0924-8579(03)00209-7. 13678823

[15] Comai L, Sen LC, Stalker DM 1983 An altered aroA gene product confers resistance to the herbicide glyphosate. Science 221:370–371. doi:10.1126/science.221.4608.370. 17798892

[16] Stalker DM, Hiatt WR, Comai L 1985 A single amino acid substitution in the enzyme 5-enolpyruvylshikimate-3-phosphate synthase confers resistance to the herbicide glyphosate. J Biol Chem 260:4724–4728. 2985565

[17] Trastoy R, Manso T, Fernández-García L, Blasco L, Ambroa A, Pérez del Molino ML, Bou G, García-Contreras R, Wood TK, Tomás M 2018 Mechanisms of bcterial tolerance and persistence in the gastrointestinal and respiratory environments. Clin Microbiol Rev 31:e00023-18. doi:10.1128/CMR.00023-18. 30068737 PMC6148185

[18] Tierney ARP, Rather PN 2019 Roles of two-component regulatory systems in antibiotic resistance. Future Microbiol 14:533–552. doi:10.2217/fmb-2019-0002. 31066586 PMC6526388

[19] Battesti A, Majdalani N, Gottesman S 2011 The RpoS-mediated general stress response in *Escherichia coli*. Annu Rev Microbiol 65:189–213. doi:10.1146/annurev-micro-090110-102946. 21639793 PMC7356644

[20] Szklarczyk D, Gable AL, Lyon D, Junge A, Wyder S, Huerta-Cepas J, Simonovic M, Doncheva NT, Morris JH, Bork P, Jensen LJ, von Mering C 2019 STRING v11: protein-protein association networks with increased coverage, supporting functional discovery in genome-wide experimental datasets. Nucleic Acids Res 47:D607–D613. doi:10.1093/nar/gky1131. 30476243 PMC6323986

[21] Pu Y, Zhao Z, Li Y, Zou J, Ma Q, Zhao Y, Ke Y, Zhu Y, Chen H, Baker MAB, Ge H, Sun Y, Xie XS, Bai F 2016 Enhanced efflux activity facilitates drug tolerance in dormant bacterial cells. Mol Cell 62:284–294. doi:10.1016/j.molcel.2016.03.035. 27105118 PMC4850422

[22] Wu Y, Vulić M, Keren I, Lewis K 2012 Role of oxidative stress in persister tolerance. Antimicrob Agents Chemother 56:4922–4926. doi:10.1128/AAC.00921-12. 22777047 PMC3421885

[23] Kurenbach B, Gibson PS, Hill AM, Bitzer AS, Silby MW, Godsoe W, Heinemann JA 2017 Herbicide ingredients change Salmonella enterica sv. Typhimurium and Escherichia coli antibiotic responses. Microbiology (Reading) 163:1791–1801. doi:10.1099/mic.0.000573. 29139345 PMC5845734

[24] Motekaitis RJ, Martell AE 1985 Metal chelate formation by N-phosphonomethylglycine and related ligands. J Coord Chem 14:139–149. doi:10.1080/00958978508073900.

[25] Banerjee R, Weisenhorn E, Schwartz KJ, Myers KS, Glasner JD, Perna NT, Coon JJ, Welch RA, Kiley J 2020 Tailoring a global iron regulon to a uropathogen. mBio 11:e00351-20. doi:10.1128/mBio.00351-20. 32209682 PMC7157518

[26] Van den Bergh B, Michiels JE, Wenseleers T, Windels EM, Vanden Boer P, Kestemont D, De Meester L, Verstrepen KJ, Verstraeten N, Fauvart M, Michiels J 2016 Frequency of antibiotic application drives rapid evolutionary adaptation of Escherichia coli persistence. Nat Microbiol 1:16020. doi:10.1038/nmicrobiol.2016.20. 27572640

[27] Roemhild R, Linkevicius M, Andersson DI 2020 Molecular mechanisms of collateral sensitivity to the antibiotic nitrofurantoin. PLoS Biol 18:e3000612. doi:10.1371/journal.pbio.3000612. 31986134 PMC7004380

[28] Gefen O, Chekol B, Strahilevitz J, Balaban NQ 2017 TDtest: easy detection of bacterial tolerance and persistence in clinical isolates by a modified disk-diffusion assay. Sci Rep 7:41284. doi:10.1038/srep41284. 28145464 PMC5286521

[29] Lucas AE, Ito R, Mustapha MM, McElheny CL, Mettus RT, Bowler SL, Kantz SF, Pacey MP, Pasculle AW, Cooper VS, Doi Y 2017 Frequency and mechanisms of spontaneous fosfomycin nonsusceptibility observed upon disk diffusion testing of Escherichia coli. J Clin Microbiol 56:e01368-17. doi:10.1128/JCM.01368-17. 29093108 PMC5744208

[30] Lenski RE 1998 Bacterial evolution and the cost of antibiotic resistance. Int Microbiol 1:265–270. 10943373

[31] Clark NA, Hafner M, Kouril M, Williams EH, Muhlich JL, Pilarczyk M, Niepel M, Sorger PK, Medvedovic M 2017 GRcalculator: an online tool for calculating and mining dose–response data. BMC Cancer 17:698. doi:10.1186/s12885-017-3689-3. 29065900 PMC5655815

[32] Hafner M, Niepel M, Chung M, Sorger PK 2016 Growth rate inhibition metrics correct for confounders in measuring sensitivity to cancer drugs. Nat Methods 13:521–527. doi:10.1038/nmeth.3853. 27135972 PMC4887336

[33] Randall LP, Cooles SW, Coldham NG, Penuela EG, Mott AC, Woodward MJ, Piddock LV, Webber MA 2007 Commonly used farm disinfectants can select for mutant Salmonella enterica serovar Typhimurium with decreased susceptibility to biocides and antibiotics without compromising virulence. J Antimicrob Chemother 60:1273–1280. doi:10.1093/jac/dkm359. 17897935

[34] Lu W, Li L, Chen M, Zhou Z, Zhang W, Ping S, Yan Y, Wang J, Lin M 2013 Genome-wide transcriptional responses of Escherichia coli to glyphosate, a potent inhibitor of the shikimate pathway enzyme 5-enolpyruvylshikimate-3-phosphate synthase. Mol Biosyst 9:522–530. doi:10.1039/c2mb25374g. 23247721

[35] Li L, Zhou Z, Jin W, Wan Y, Lu W 2015 A transcriptomic analysis for identifying the unintended effects of introducing a heterologous glyphosate-tolerant EPSP synthase into Escherichia coli. Mol Biosyst 11:852–858. doi:10.1039/c4mb00566j. 25564113

[36] Tincher C, Long H, Behringer MG, Walker N, Lynch M 2017 The glyphosate-based herbicide Roundup does not elevate genome-wide mutagenesis of Escherichia coli. G3 Genes Genomes Genetics 7:3331–3335. doi:10.1534/g3.117.300133. 28983068 PMC5633383

[37] Gutierrez A, Laureti L, Crussard S, Abida H, Rodríguez-Rojas A, Blázquez J, Baharoglu Z, Mazel D, Darfeuille F, Vogel J, Matic I 2013 β-Lactam antibiotics promote bacterial mutagenesis via an RpoS-mediated reduction in replication fidelity. Nat Commun 4:1610. doi:10.1038/ncomms2607. 23511474 PMC3615471

[38] Coil D, Jospin G, Darling AE 2015 A5-miseq: an updated pipeline to assemble microbial genomes from Illumina MiSeq data. Bioinformatics 31:587–589. doi:10.1093/bioinformatics/btu661. 25338718

[39] Seemann T 2014 Prokka: rapid prokaryotic genome annotation. Bioinformatics 30:2068–2069. doi:10.1093/bioinformatics/btu153. 24642063

[40] Li H 2013 Aligning sequence reads, clone sequences and assembly contigs with BWA-MEM. arXiv:1303.3997 https://arxiv.org/abs/1303.3997.

[41] Garrison E, Marth G 2012 Haplotype-based variant detection from short-read sequencing. arXiv:1207.3907 https://arxiv.org/abs/1207.3907.

[42] Deatherage DE, Barrick JE 2014 Identification of mutations in laboratory-evolved microbes from next-generation sequencing data using breseq. Methods Mol Biol 1151:165–188. doi:10.1007/978-1-4939-0554-6_12. 24838886 PMC4239701

[43] Wareth G, Eravci M, Weise C, Roesler U, Melzer F, Sprague LD, Neubauer H, Murugaiyan J 2016 Comprehensive identification of immunodominant proteins of Brucella abortus and Brucella melitensis using antibodies in the sera from naturally infected hosts. Int J Mol Sci 17:659. doi:10.3390/ijms17050659. PMC4881485 27144565

[44] Cox J, Hein MY, Luber CA, Paron I, Nagaraj N, Mann M 2014 Accurate proteome-wide label-free quantification by delayed normalization and maximal peptide ratio extraction, termed MaxLFQ. Mol Cell Proteomics 13:2513–2526. doi:10.1074/mcp.M113.031591. 24942700 PMC4159666

[45] Tyanova S, Temu T, Sinitcyn P, Carlson A, Hein MY, Geiger T, Mann M, Cox J 2016 The Perseus computational platform for comprehensive analysis of (prote)omics data. Nat Methods 13:731–740. doi:10.1038/nmeth.3901. 27348712

[46] Perez-Riverol Y, Csordas A, Bai J, Bernal-Llinares M, Hewapathirana S, Kundu DJ, Inuganti A, Griss J, Mayer G, Eisenacher M, Pérez E, Uszkoreit J, Pfeuffer J, Sachsenberg T, Yılmaz Ş, Tiwary S, Cox J, Audain E, Walzer M, Jarnuczak AF, Ternent T, Brazma A, Vizcaíno JA 2019 The PRIDE database and related tools and resources in 2019: improving support for quantification data. Nucleic Acids Res 47:D442–D450. doi:10.1093/nar/gky1106. 30395289 PMC6323896

[47] Funke T, Yang Y, Han H, Healy-Fried M, Olesen S, Becker A, Schönbrunn E 2009 Structural basis of glyphosate resistance resulting from the double mutation Thr97-Ile and Pro101-Ser in 5-enolpyruvylshikimate-3-phosphate synthase from Escherichia coli. J Biol Chem 284:9854–9860. doi:10.1074/jbc.M809771200. 19211556 PMC2665107

