# Supplementary material for: Selection for Resistance to a Glyphosate-Containing Herbicide in Salmonella enterica Does Not Result in a Sustained Activation of the Tolerance Response or Increased Cross-Tolerance and Cross-Resistance to Clinically Important Antibiotics
Source: Appl Environ Microbiol. 2020 Nov 24;86(24):e01204-20. doi: 10.1128/AEM.01204-20 (PMC7688225; doi:10.1128/AEM.01204-20)
Supplement: Supplemental file 1 [file AEM.01204-20-s0001.pdf]

Supplementary material

Supplementary figure 1. Volcano plots of the entire proteomes of the four ancestral isolates (A) of *Salmonella enterica* Enteritidis (Se) and Typhimurium (St) challenged with GBH Roundup LB plus (RU) versus non-challenged counterparts. Panels of volcano plots were generated using the PERSEUS software by comparison of groups A_RU vs A of all compared isolates. Proteins were quantified using label free quantitative mass spectrometry. Proteins were graphed by Log2 fold change (Difference) and significance (-Log p) using a false discovery rate of 0.05. Most important proteins were labeled.

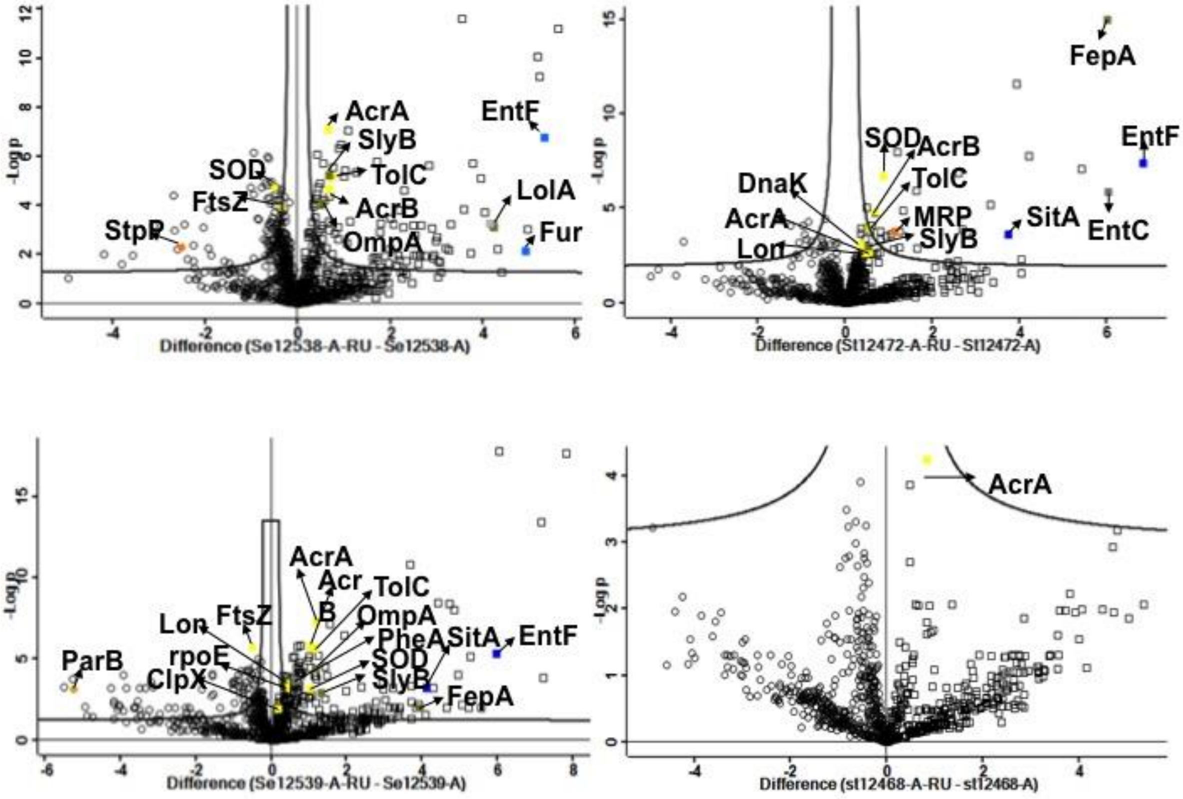

Supplementary figure 2. Volcano plots of the entire proteomes of the three GBH Roundup LB plus (RU) resistant mutants (M) versus ancestors (A) of *Salmonella enterica* Enteritidis (Se) and Typhimurium (St) in the absence of the herbicide (constitutive expression). Panels of volcano plots were generated using the PERSEUS software by comparison of groups M vs A. Proteins were quantified using label free quantitative mass spectrometry. Proteins were graphed by Log2 fold change (Difference) and significance (-Log p) using a false discovery rate of 0.05. Most important proteins were labeled.

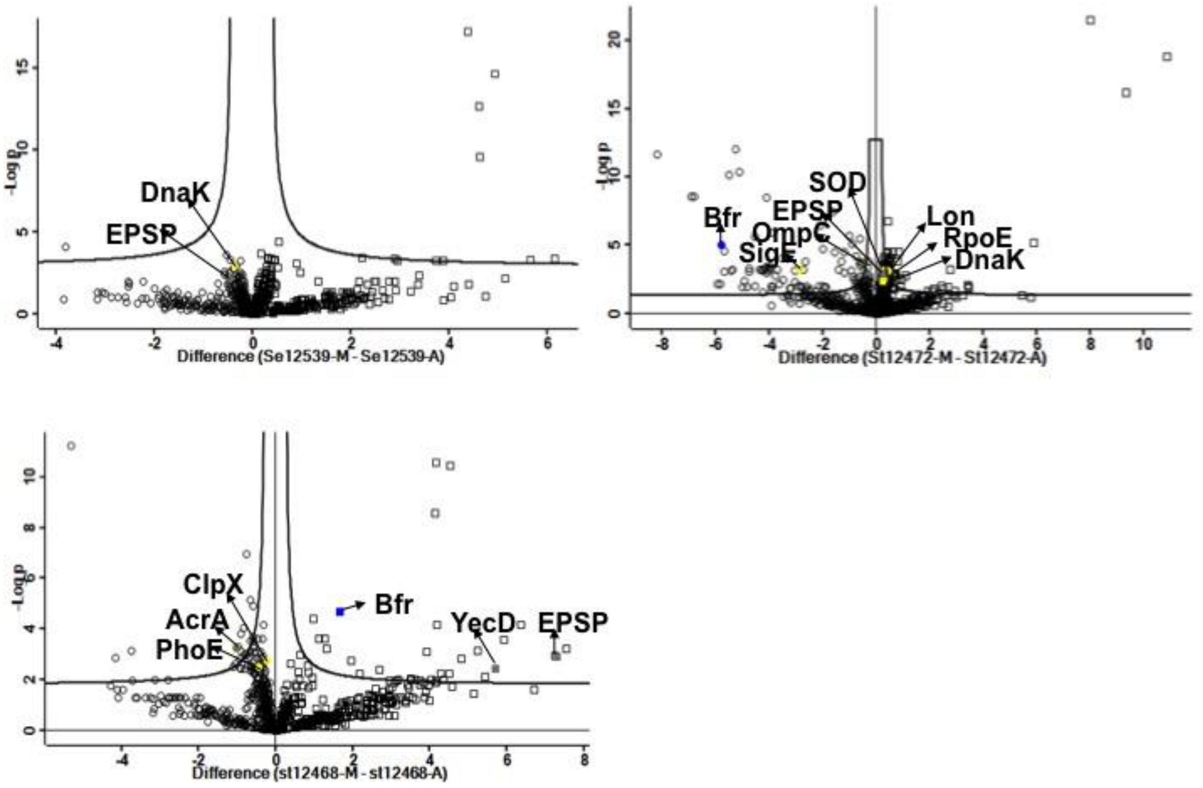

Supplementary figure 3. Results of the TDtest with cefepim (FEP), ceftazidime (CAZ), rifampicin (RIF), colisitin (CT) and tigecyclin (TGC) in the absence of exposure to GBH Roundup LB plus (RU) in the resistant mutants and their ancestors. Note the absence of tolerant colonies within the clearance zones.

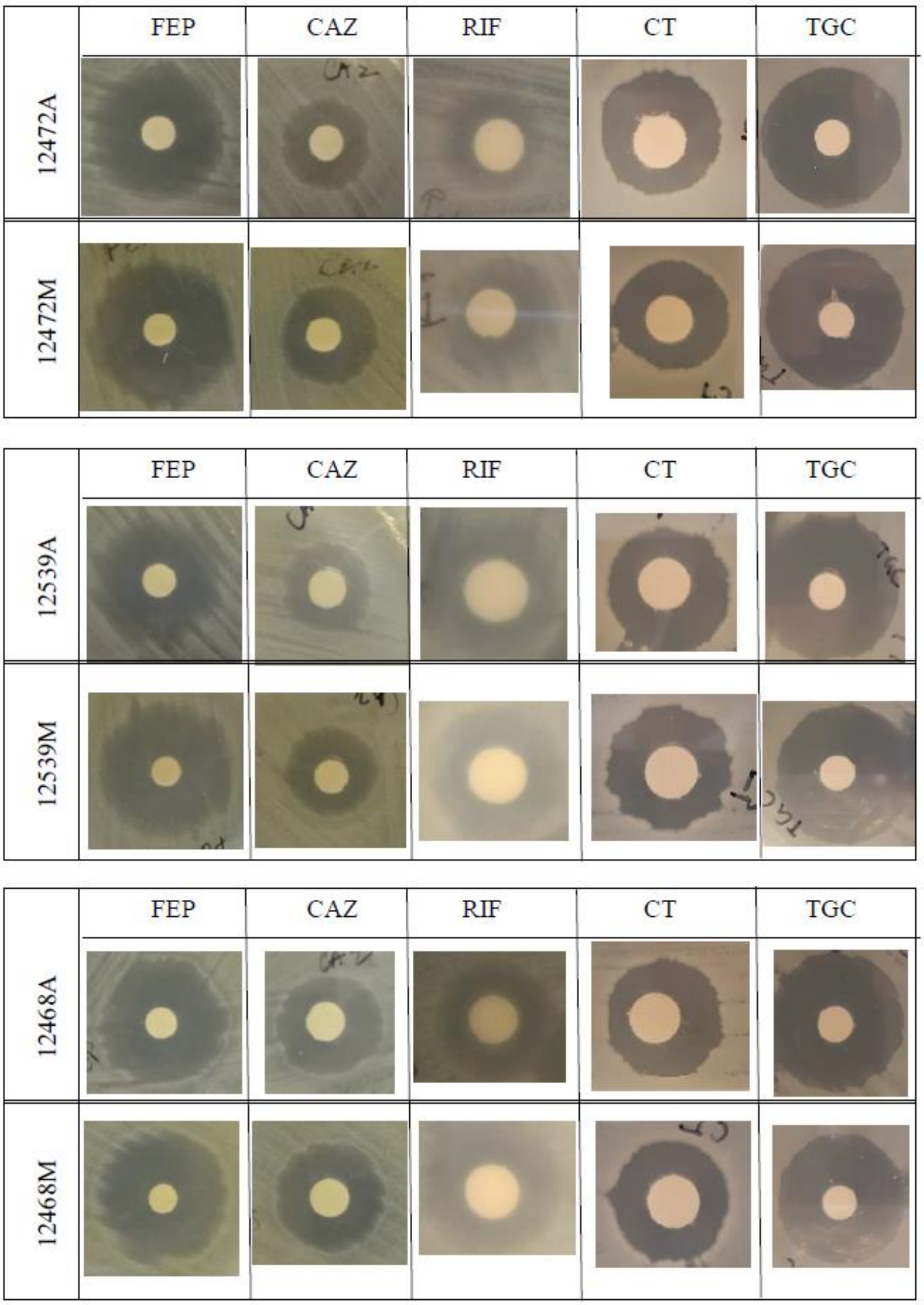

Supplementary figure 4. Results of the TDtest with Fosfomycin in the absence of exposure to GBH Roundup LB plus (RU) in the resistant mutants and their ancestors. Colonies that appeared within the clearance zone were moderately resistant to Fosfomycin as determined by MIC and represent spontaneous mutants.

	**12539**
**Ancestor**	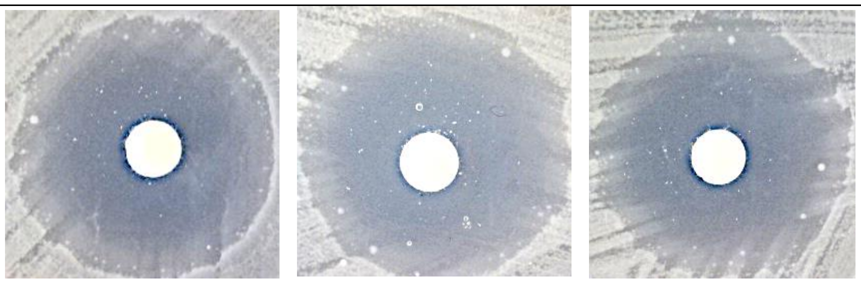
**Mutant**	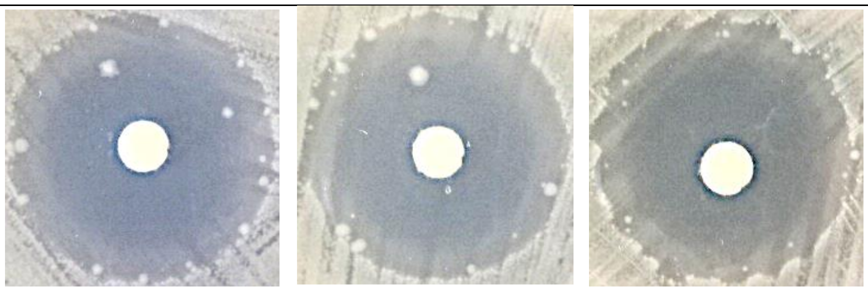

	**12468**
**Ancestor**	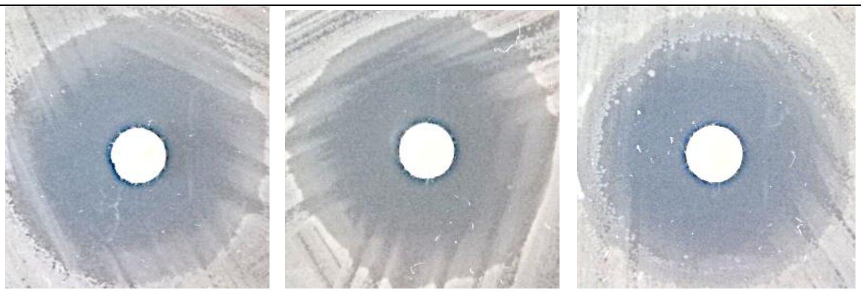
**Mutant**	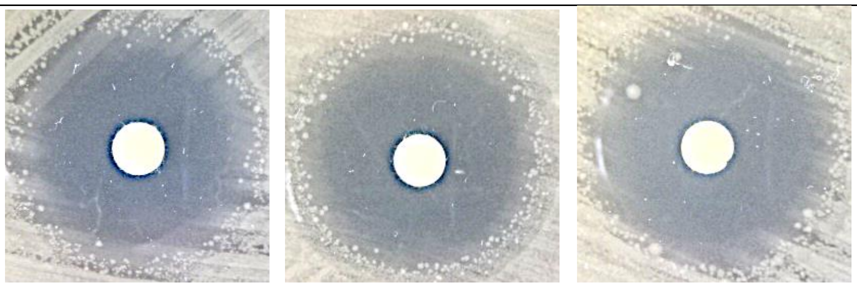

	**12472**

**Ancestor**	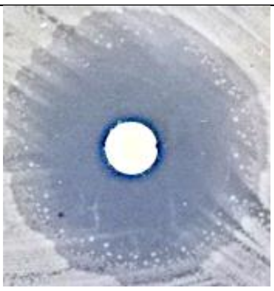 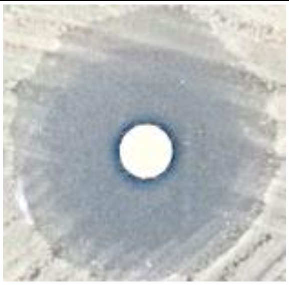 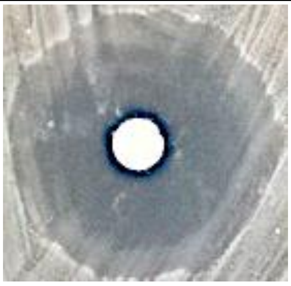
**Mutant**	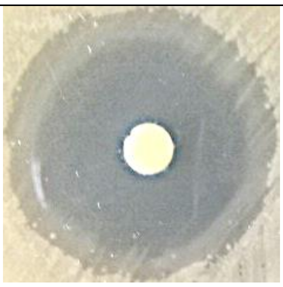 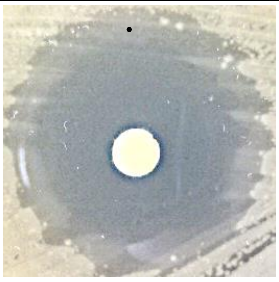 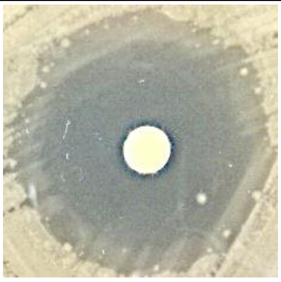

Supplementary figure 5. Box plots of fitness costs calculated with GRcalculator. The figure shows box plot of log 10 of GR50, GRmax and GRinf for the three isolates (S.Typhimurium 12468; S.Typhimurium 12472; S. Enteritidis 12539) calculated with GR calculator. Comparison made of four technical replicates of each ancestral (red) and mutation (green) strain for 12468. Statistical significance above the bracket.

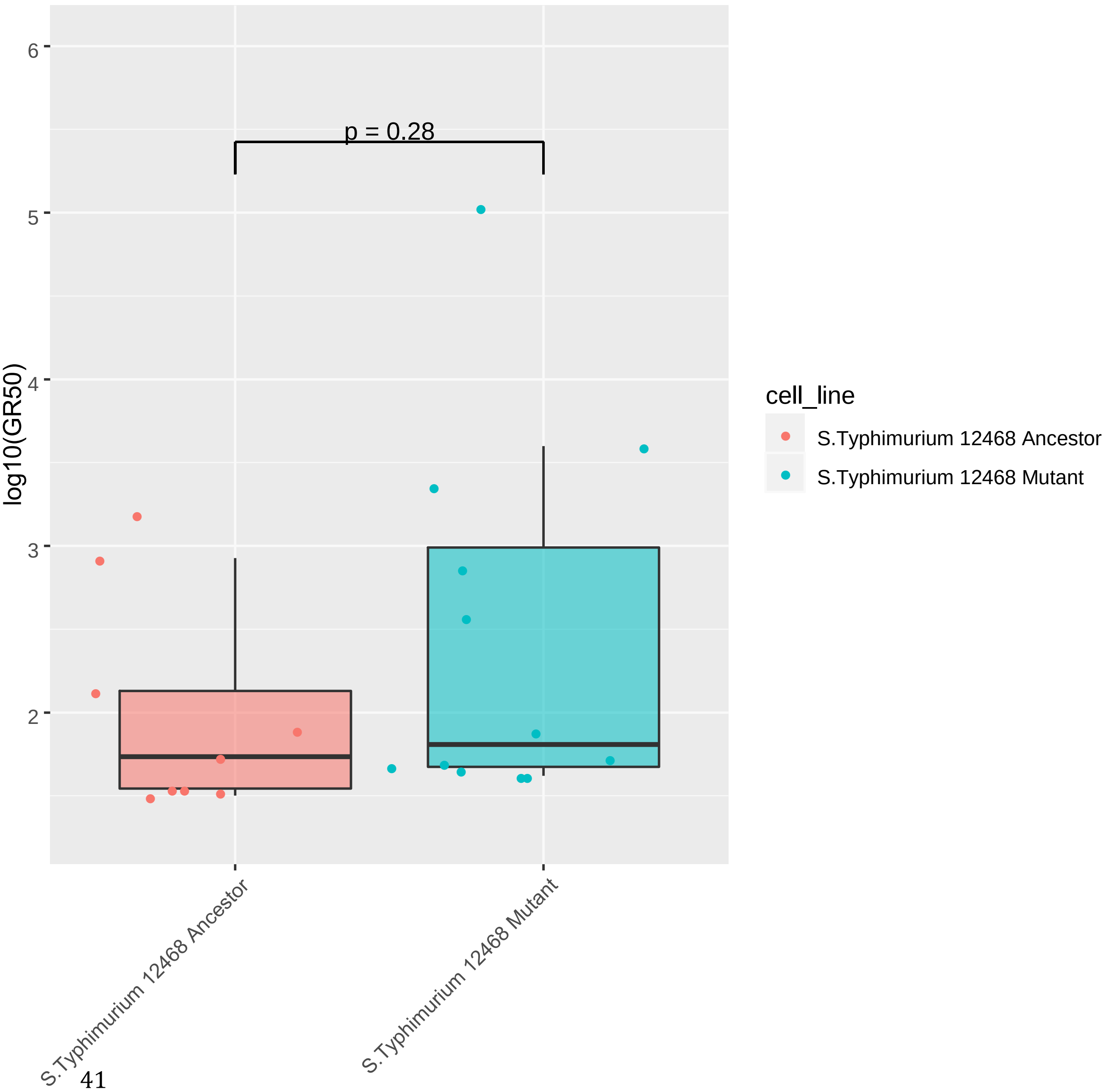

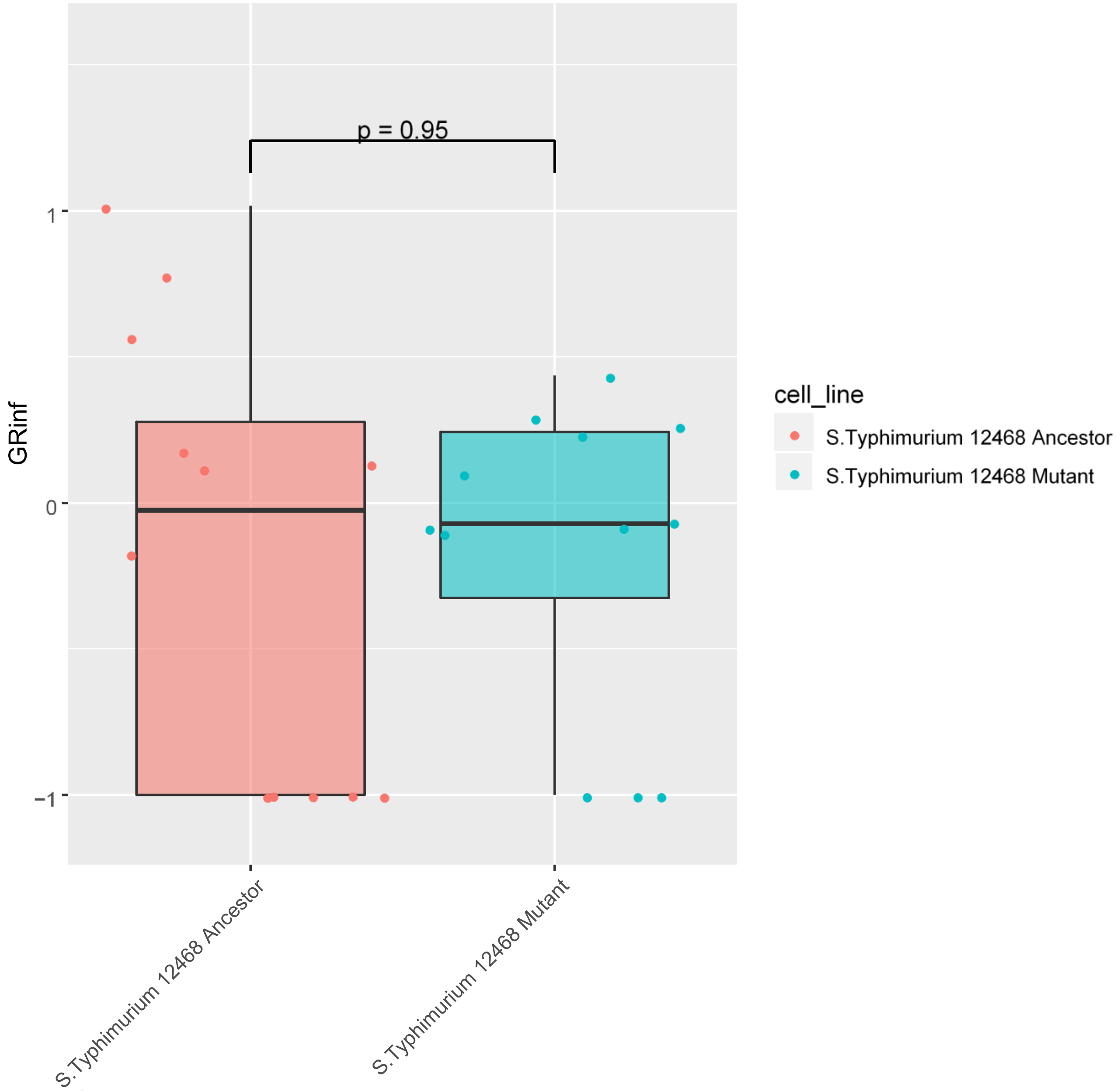
p = 0.95
GRinf
1
0
−1
S.Typhimurium 12468 Ancestor
S.Typhimurium 12468 Mutant
cell_line
S.Typhimurium 12468 Ancestor
S.Typhimurium 12468 Mutant
42

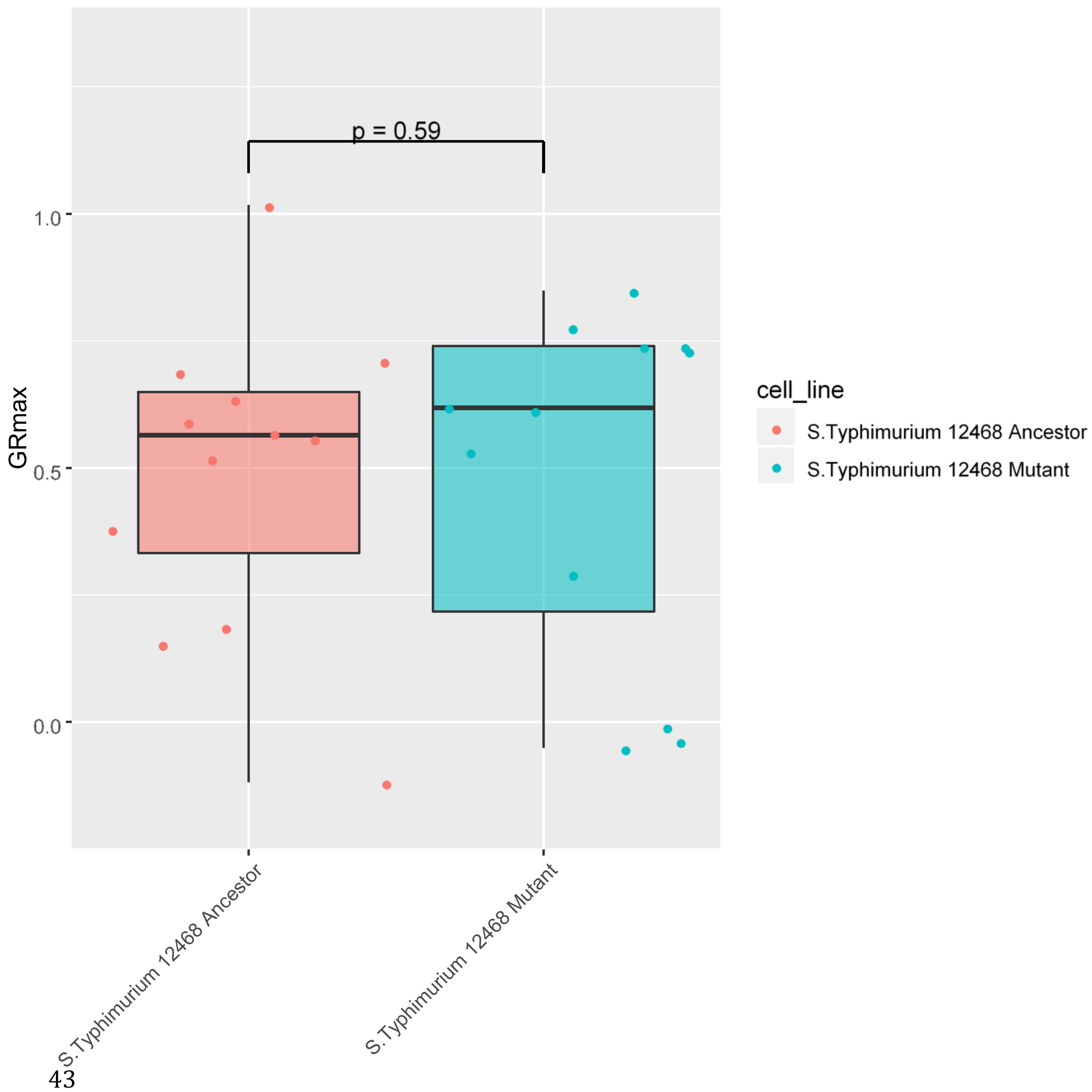
p = 0.59
GRmax
1.0
0.5
0.0
S.Typhimurium 12468 Ancestor
S.Typhimurium 12468 Mutant
cell_line
S.Typhimurium 12468 Ancestor
S.Typhimurium 12468 Mutant

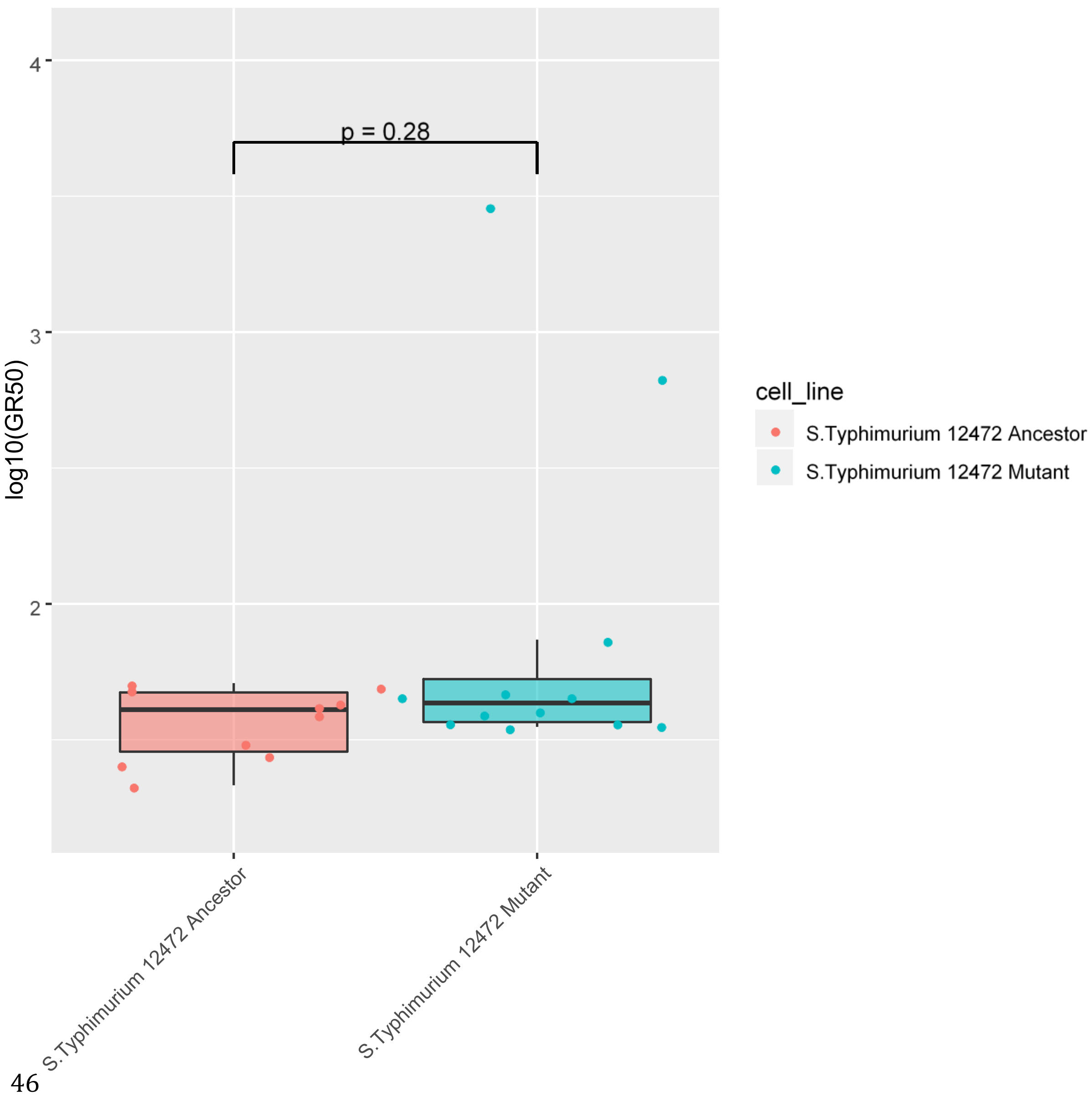
p = 0.28
log10(GR50)
4
3
2
S.Typhimurium 12472 Ancestor
S.Typhimurium 12472 Mutant
cell_line
S.Typhimurium 12472 Ancestor
S.Typhimurium 12472 Mutant

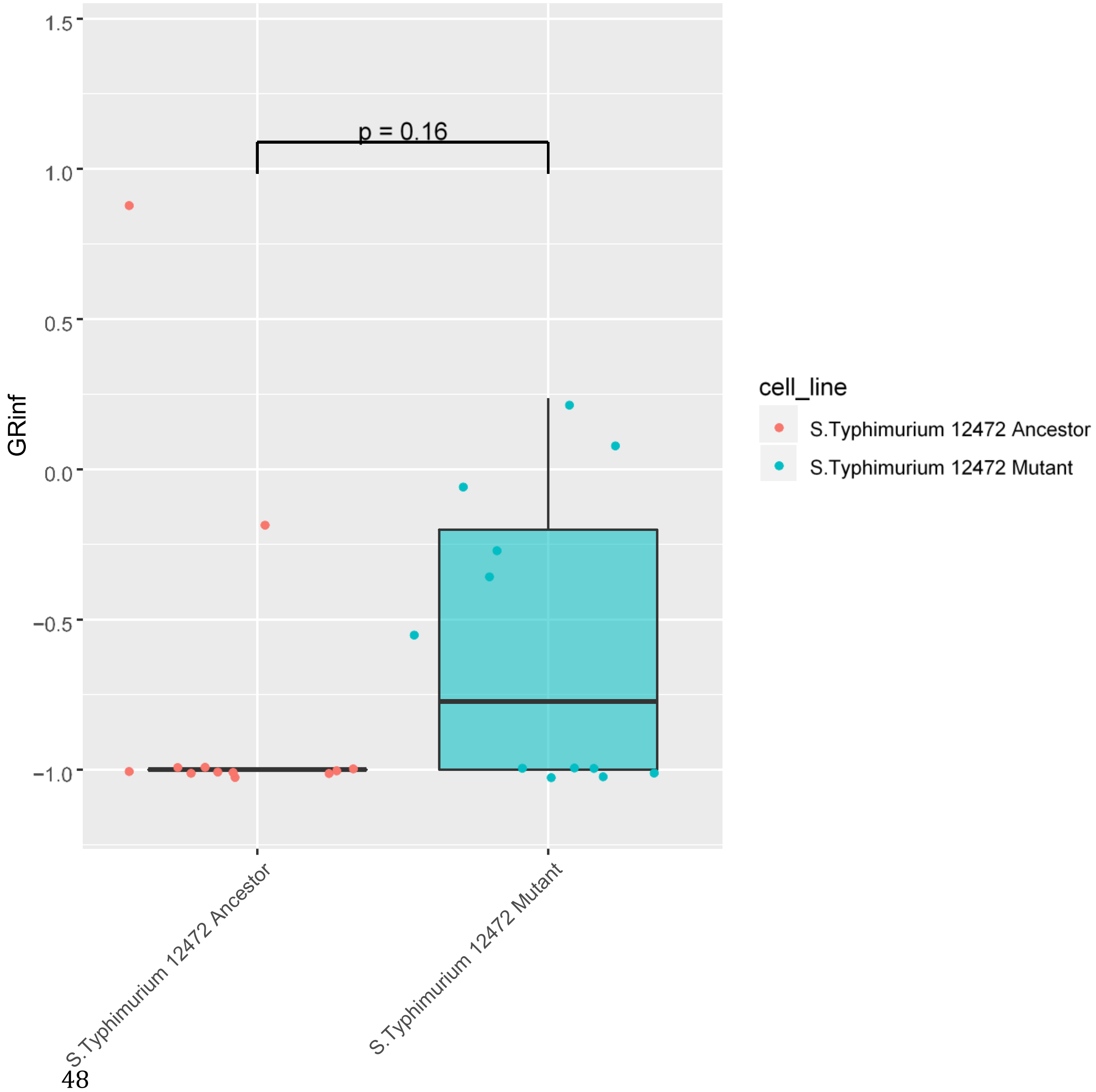
p = 0.16
GRinf
1.5
1.0
0.5
0.0
−0.5
−1.0
S.Typhimurium 12472 Ancestor
S.Typhimurium 12472 Mutant
cell_line
S.Typhimurium 12472 Ancestor
S.Typhimurium 12472 Mutant

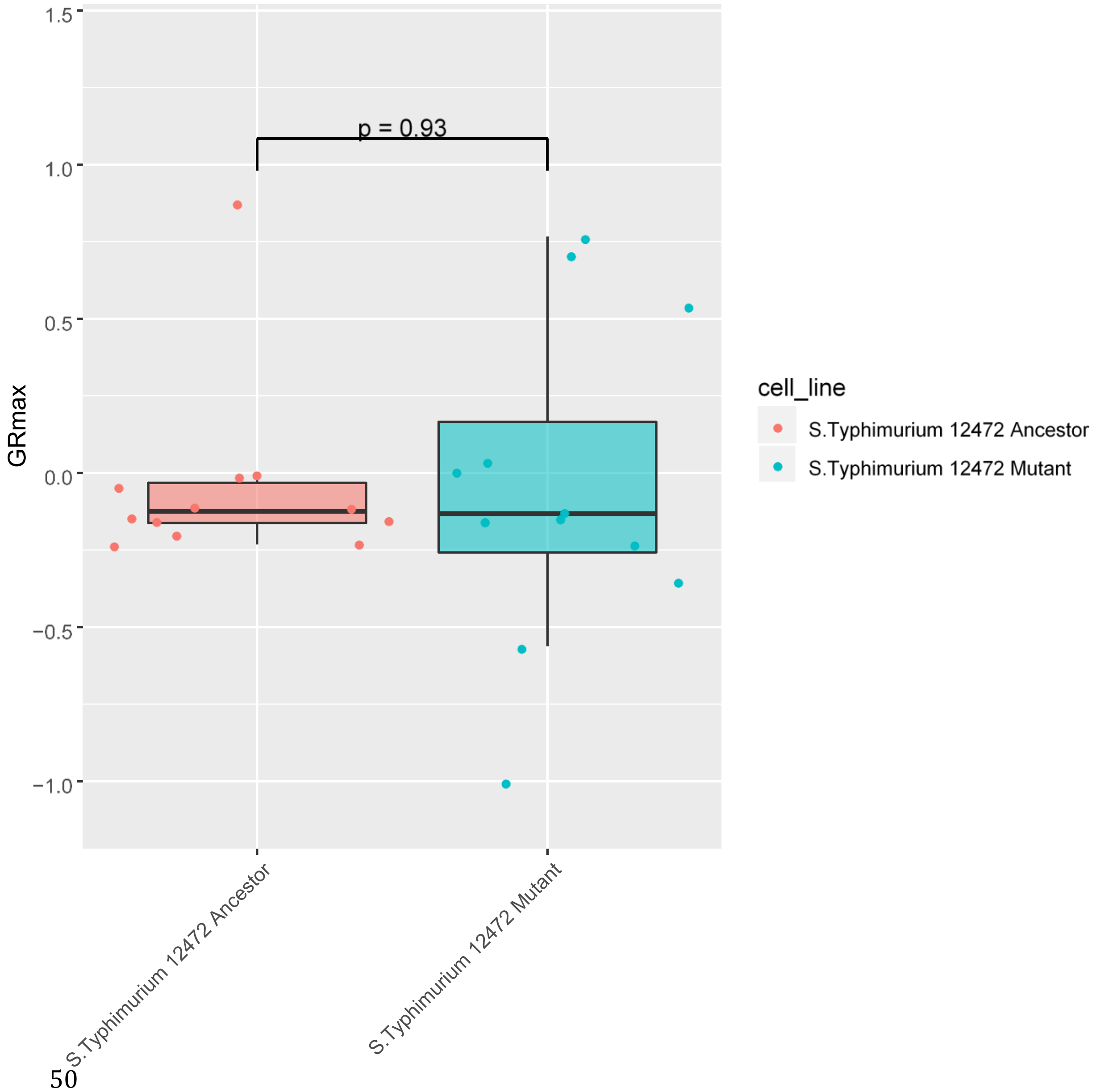
p = 0.93
GRmax
1.5
1.0
0.5
0.0
−0.5
−1.0
S.Typhimurium 12472 Ancestor
S.Typhimurium 12472 Mutant
cell_line
S.Typhimurium 12472 Ancestor
S.Typhimurium 12472 Mutant

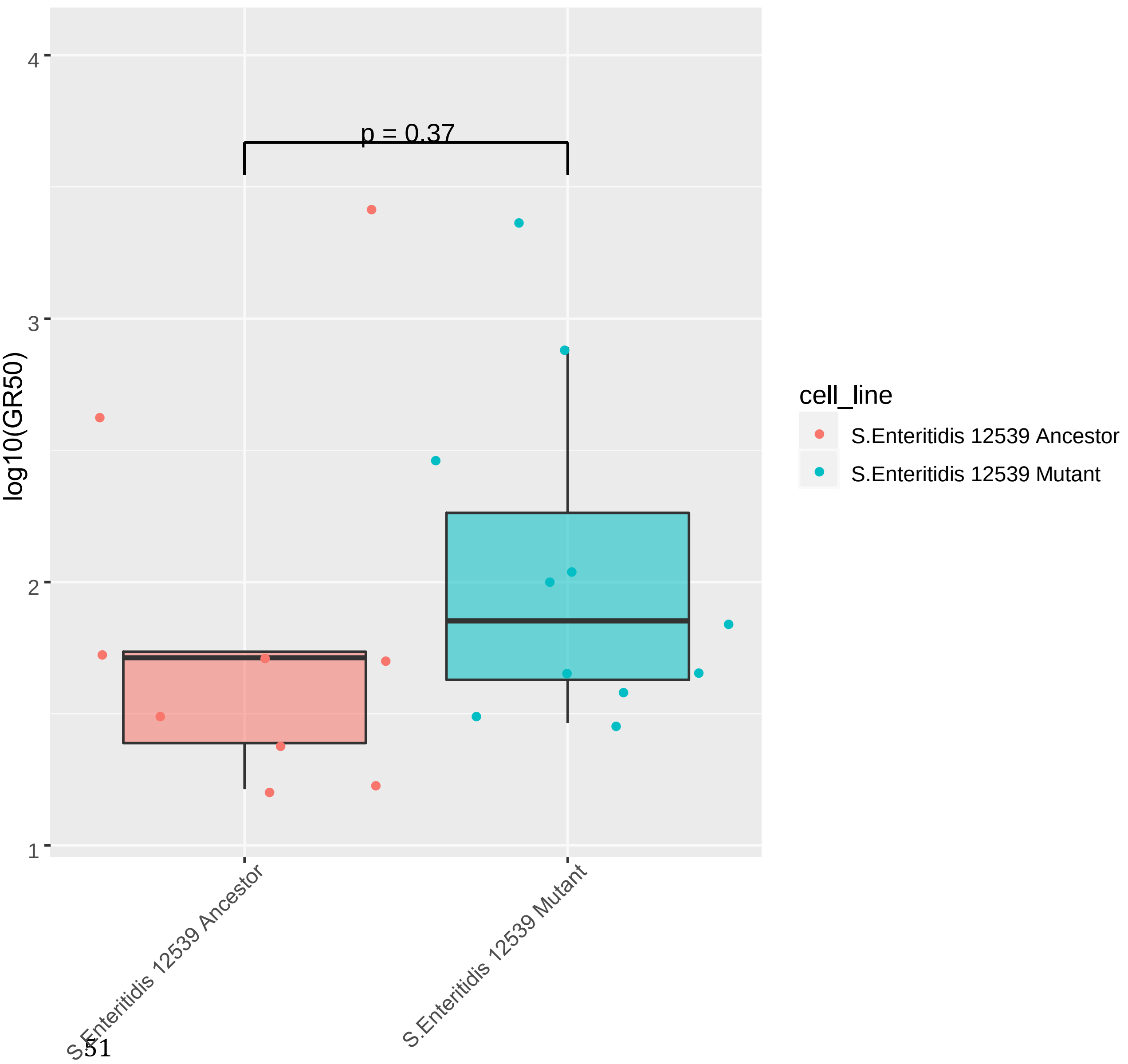
p = 0.37
log10(GR50)
4
3
2
1
S.Enteritidis 12539 Ancestor
S.Enteritidis 12539 Mutant
cell_line
S.Enteritidis 12539 Ancestor
S.Enteritidis 12539 Mutant

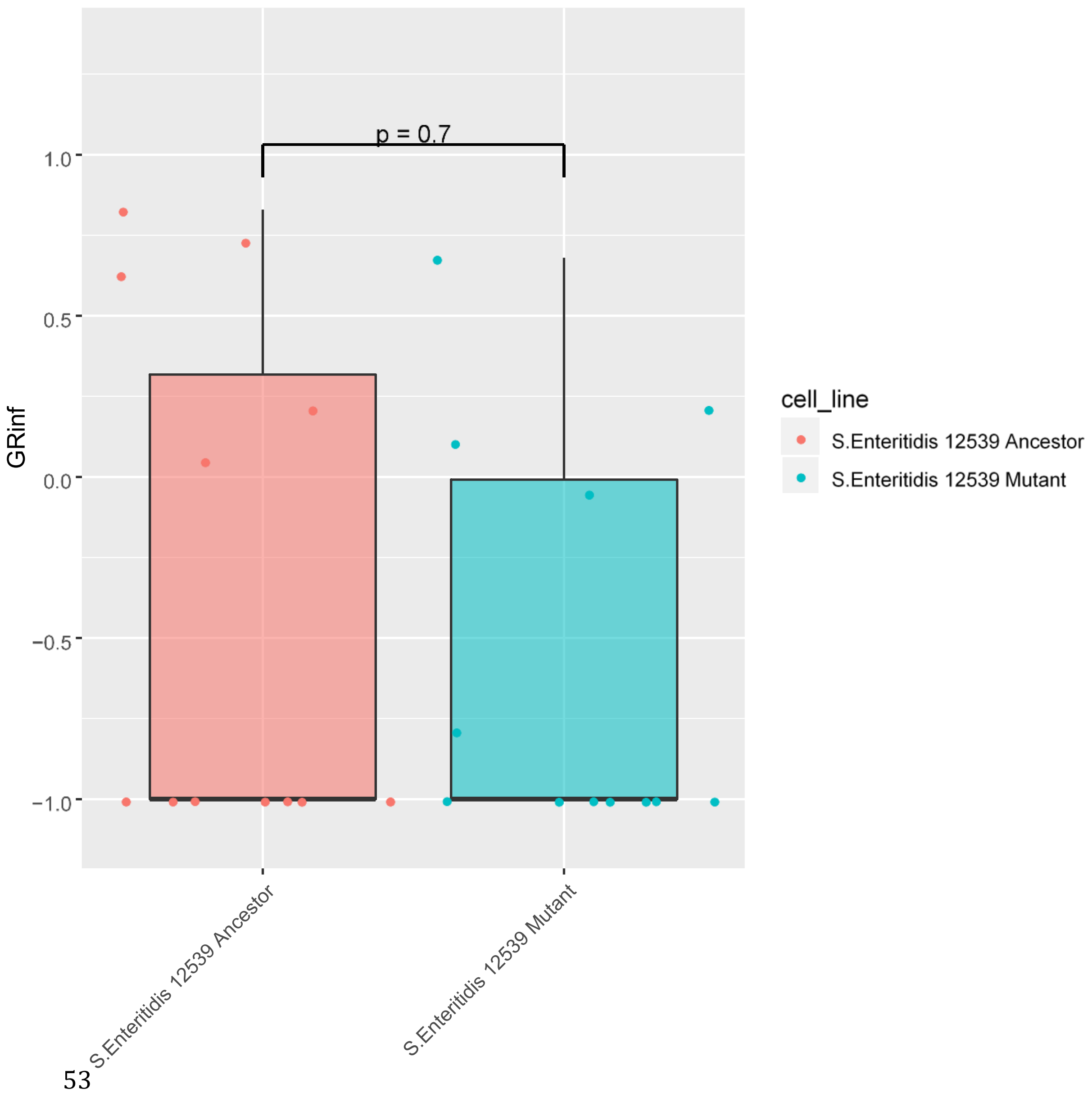
p = 0.7
1.0
0.5
0.0
−0.5
−1.0
GRinf
cell_line
S.Enteritidis 12539 Ancestor
S.Enteritidis 12539 Mutant
S.Enteritidis 12539 Ancestor
S.Enteritidis 12539 Mutant

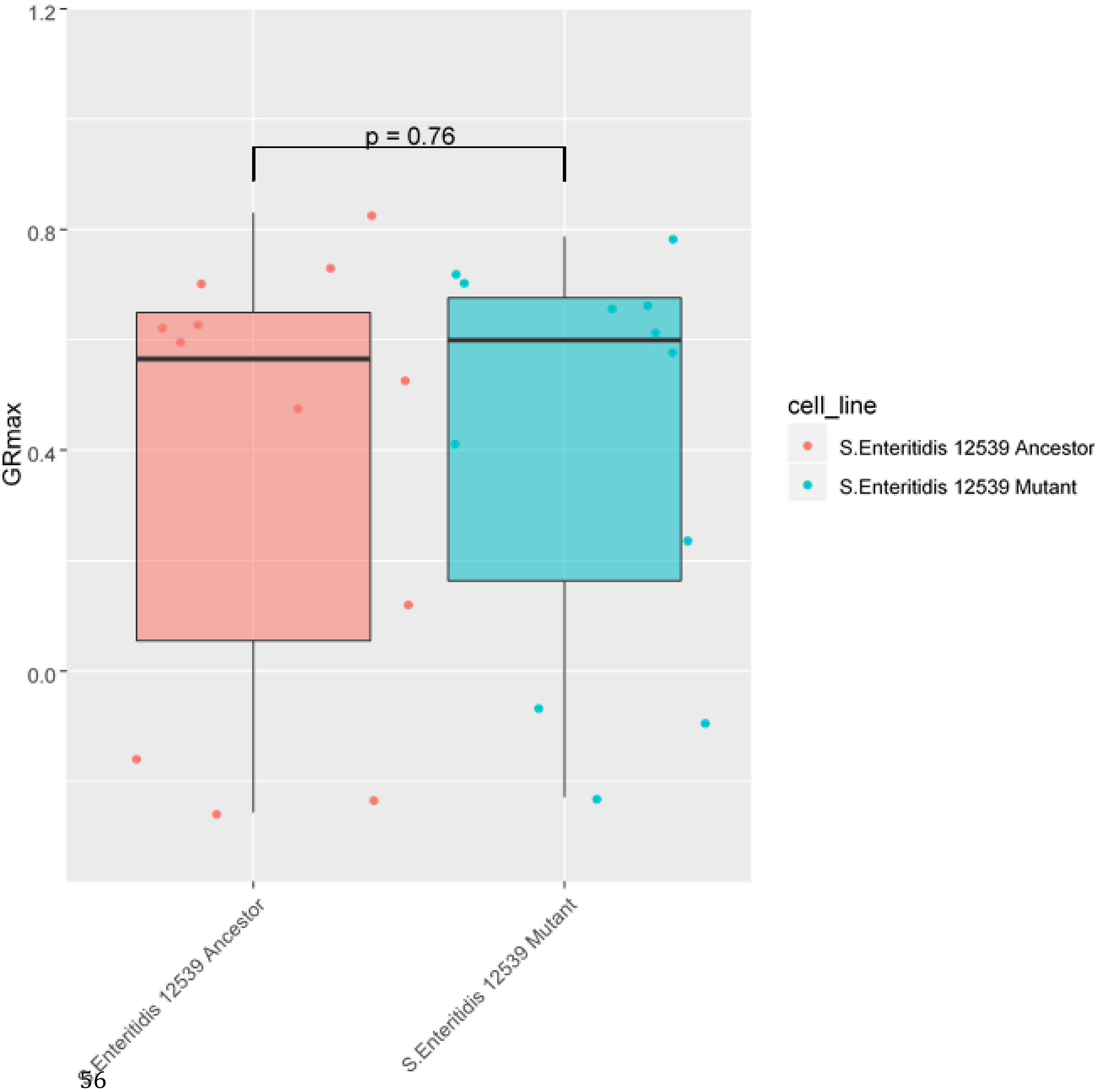
p = 0.76
GRmax
1.2
0.8
0.4
0.0
cell_line
S.Enteritidis 12539 Ancestor
S.Enteritidis 12539 Mutant
S.Enteritidis 12539 Ancestor
S.Enteritidis 12539 Mutant

Supplementary table 2. Complete list of significantly differentially expressed proteins. Global label-free quantitative proteomics analysis of the resistant mutants (M) and their ancestors (A) in the presence and absence of sub-inhibitory (1/4 x MIC) concentrations of glyphosate-based herbicide (GBH) Roundup LB Plus (RU).

**A RU vs A constitutive**			
S. enteritidis 12538			
**Strain**	**Protein ID/FASTA Head**	**Protein Name**	**Expression**
S. enteritidis 12538	OKIFFGJM_00295	potassium-transportingATPasesubunitB	5,6
S. enteritidis 12538	OKIFFGJM_00176	enterobactinsynthetasecomponentF	5,3
S. enteritidis 12538	OKIFFGJM_02662	ABCtransportATP-bindingsubunit	5,2
S. enteritidis 12538	OKIFFGJM_00900	glycinebetaine-bindingperiplasmicprotein	5,2
S. enteritidis 12538	OKIFFGJM_03121	DNAprotectionduringstarvationprotein	5,0
S. enteritidis 12538	OKIFFGJM_00282	ferricuptakeregulationprotein	4,9
S. enteritidis 12538	OKIFFGJM_00050	ATP-dependent Clpproteaseproteolyticsubunit	4,3
S. enteritidis 12538	OKIFFGJM_04152	outer-membranelipoproteincarrierprotein	4,2
S. enteritidis 12538	OKIFFGJM_00902	glycinebetaine/L-prolinetransportATP-bindingprotein	4,2
S. enteritidis 12538	OKIFFGJM_00183	isochorismatesynthaseEntC	4,2
S. enteritidis 12538	OKIFFGJM_00283	Flavodoxin1	4,0
S. enteritidis 12538	OKIFFGJM_00849	irontransportperiplasmic-bindingprotein	4,0
S. enteritidis 12538	OKIFFGJM_02456	NicotinamidasefamilyproteinYcaC	3,8
S. enteritidis 12538	OKIFFGJM_00747	putativeflavoproteinYqcA(clusteredwith tRNApseudouridinesynthaseC)	3,7
S. enteritidis 12538	OKIFFGJM_02274	3-isopropylmalatedehydratase	3,6
S. enteritidis 12538	OKIFFGJM_01353	membrane-boundbeta-hydroxylase	3,5
S. enteritidis 12538	OKIFFGJM_02663	Iron-sulfurclusterassemblyproteinSufB	3,3

S. enteritidis 12538	OKIFFGJM_04187	putativemetal-bindingenzymeYcbL-likeprotein	3,3
S. enteritidis 12538	OKIFFGJM_04059	Anti-adapterproteiniraP	3,2
S. enteritidis 12538	OKIFFGJM_00184	enterobactinsynthasesubunitE	3,0
S. enteritidis 12538	OKIFFGJM_00186	2,3-dihydro-2,3-dihydroxybenzoatedehydrogenase	3,0
S. enteritidis 12538	OKIFFGJM_03166	6-phosphogluconolactonase	2,9
S. enteritidis 12538	OKIFFGJM_01992	NADHdehydrogenaseoperontranscriptionalregulator	2,9
S. enteritidis 12538	OKIFFGJM_01322	acidphosphatase	2,8
S. enteritidis 12538	OKIFFGJM_01101	phosphoglyceroltransferase	2,8
S. enteritidis 12538	OKIFFGJM_00777	ATPsulfurylase	2,7
S. enteritidis 12538	OKIFFGJM_04689	shikimate5-dehydrogenase	2,6
S. enteritidis 12538	OKIFFGJM_02252	UDP-3-O-[3-hydroxymyristoyl]N-acetylglucosaminedeacetylase	2,6
S. enteritidis 12538	OKIFFGJM_03347	curlingenestranscriptionalactivator	2,5
S. enteritidis 12538	OKIFFGJM_02721	rhamnosyltransferase	2,5
S. enteritidis 12538	OKIFFGJM_02660	bifunctionalcysteinedesulfurase/selenocysteinelyase	2,5
S. enteritidis 12538	OKIFFGJM_02417	glucosyltransferase	2,3
S. enteritidis 12538	OKIFFGJM_01994	oxetanocinAbiosyntheticenzyme	2,3
S. enteritidis 12538	OKIFFGJM_02273	3-isopropylmalatedehydrogenase	2,2
S. enteritidis 12538	OKIFFGJM_03842	periplasmicsulfatebindingprotein	2,2
S. enteritidis 12538	OKIFFGJM_00302	Allophanatehydrolase2subunit2	2,1
S. enteritidis 12538	OKIFFGJM_00099	LeadcadmiumzincandmercurytransportingATPase	2,0
S. enteritidis 12538	OKIFFGJM_02110	thiosulfate-bindingprotein	2,0
S. enteritidis 12538	OKIFFGJM_02983	Leu/Ile/Val/Thr-bindingprotein;leucine-specificbindingprotein	1,9
S. enteritidis 12538	OKIFFGJM_02171	[Protein-PII]uridylyltransferase	1,8
S. enteritidis 12538	OKIFFGJM_00185	isochorismatase	1,8
S. enteritidis 12538	OKIFFGJM_00166	dihydropteridinereductase	1,7
S. enteritidis 12538	OKIFFGJM_02176	serineendoprotease	1,3

S. enteritidis 12538	OKIFFGJM_02096	cysteinesynthaseA	1,1
S. enteritidis 12538	OKIFFGJM_03843	CDP-diacylglycerolpyrophosphatase	1,1
S. enteritidis 12538	OKIFFGJM_01467	aconitatehydratase1	1,1
S. enteritidis 12538	OKIFFGJM_02920	osmolarityresponseregulator	1,0
S. enteritidis 12538	OKIFFGJM_00897	multidrugresistancesecretionprotein	1,0
S. enteritidis 12538	OKIFFGJM_01899	NADP-specificglutamatedehydrogenase	0,9
S. enteritidis 12538	OKIFFGJM_03748	branched-chainaminoacidaminotransferase	0,9
S. enteritidis 12538	OKIFFGJM_00105	Thioredoxindomain-containingproteinEC-YbbN	0,9
S. enteritidis 12538	OKIFFGJM_03013	glutathionereductase	0,9
S. enteritidis 12538	OKIFFGJM_03041	ATP-dependentClpproteaseATP-bindingsubunit	0,8
S. enteritidis 12538	OKIFFGJM_00361	cytochromedubiquinoloxidasesubunitIII	0,8
S. enteritidis 12538	OKIFFGJM_00523	outermembraneproteinTolC	0,8
S. enteritidis 12538	OKIFFGJM_03754	ketol-acidreductoisomerase	0,7
S. enteritidis 12538	OKIFFGJM_03749	dihydroxyaciddehydratase	0,7
S. enteritidis 12538	OKIFFGJM_01841	isocitratedehydrogenase	0,7
S. enteritidis 12538	OKIFFGJM_02587	outermembranelipoproteinSlyB	0,7
S. enteritidis 12538	OKIFFGJM_04442	sugarphosphatephosphatase	0,7
S. enteritidis 12538	OKIFFGJM_00075	acriflavinresistanceproteinB	0,7
S. enteritidis 12538	OKIFFGJM_00076	acriflavinresistanceproteinA;acriflavinresistanceproteinE	0,7
S. enteritidis 12538	OKIFFGJM_02692	glutathioneperoxidase/vitaminB12transportperiplasmicproteinBtuE	0,6
S. enteritidis 12538	OKIFFGJM_02228	bifunctionalaconitatehydratase2/2-methylisocitratedehydratase	0,6
S. enteritidis 12538	OKIFFGJM_03897	phosphoenolpyruvatecarboxylase	0,5
S. enteritidis 12538	OKIFFGJM_04062	pyrroline-5-carboxylatereductase	0,5
S. enteritidis 12538	OKIFFGJM_02195	ferrichromeoutermembranetransporter	0,5
S. enteritidis 12538	OKIFFGJM_01675	outermembraneproteinA	0,5
S. enteritidis 12538	OKIFFGJM_04080	chorismatemutase-P/prephenatedehydratase	0,5

S. enteritidis 12538	OKIFFGJM_00551	biopolymertransportproteinExbB	0,5
S. enteritidis 12538	OKIFFGJM_00786	lipoproteinNlpD	0,5
S. enteritidis 12538	OKIFFGJM_00742	Decarboxylasefamilyprotein	0,5
S. enteritidis 12538	OKIFFGJM_00398	putativeABCtransporterauxiliarycomponentYrbC	0,5
S. enteritidis 12538	OKIFFGJM_04048	delta-aminolevulinicaciddehydratase	0,5
S. enteritidis 12538	OKIFFGJM_01960	UDP-4-amino-4-deoxy-L-arabinose--oxoglutarateaminotransferase	0,5
S. enteritidis 12538	OKIFFGJM_00258	phosphatestarvation-inducibleprotein,ATP-binding	0,5
S. enteritidis 12538	OKIFFGJM_03010	oligopeptidaseA	0,4
S. enteritidis 12538	OKIFFGJM_00980	malatedehydrogenase	0,4
S. enteritidis 12538	OKIFFGJM_03859	triosephosphateisomerase	0,4
S. enteritidis 12538	OKIFFGJM_02172	2,3,4,5-tetrahydropyridine-2,6-carboxylateN-succinyltransferase	0,4
S. enteritidis 12538	OKIFFGJM_02317	carbamoyl-phosphatesynthaselargechain	0,4
S. enteritidis 12538	OKIFFGJM_00360	serineprotease	0,4
S. enteritidis 12538	OKIFFGJM_01997	helix-turn-helixdomain-containingprotein	0,4
S. enteritidis 12538	OKIFFGJM_00318	citratesynthase	0,4
S. enteritidis 12538	OKIFFGJM_01663	aminopeptidaseN	0,4
S. enteritidis 12538	OKIFFGJM_01277	FtsHproteaseregulatorHflC	0,3
S. enteritidis 12538	OKIFFGJM_00893	gamma-glutamylcysteinesynthetase	0,3
S. enteritidis 12538	OKIFFGJM_03230	NifU-likeprotein	0,3
S. enteritidis 12538	OKIFFGJM_02523	3-hydroxyaciddehydrogenase	0,3
S. enteritidis 12538	OKIFFGJM_03229	L-cysteinedesulfurase	0,3
S. enteritidis 12538	OKIFFGJM_02232	dihydrolipoamidedehydrogenase	0,3
S. enteritidis 12538	OKIFFGJM_03236	peptidaseB	0,3
S. enteritidis 12538	OKIFFGJM_02607	riboflavinsynthasealphachain	0,3
S. enteritidis 12538	OKIFFGJM_00324	2-oxoglutaratedehydrogenase E1component	0,2
S. enteritidis 12538	OKIFFGJM_01035	transaldolaseB	0,2

S. enteritidis 12538	OKIFFGJM_01464	DNAtopoisomerase1	0,2
S. enteritidis 12538	OKIFFGJM_02234	pyruvatedehydrogenaseE1component	0,2
S. enteritidis 12538	OKIFFGJM_00469	L-serinedehydratase	-4,2
S. enteritidis 12538	OKIFFGJM_02720	mannose-1-phosphateguanyltransferase	-3,6
S. enteritidis 12538	OKIFFGJM_04160	pyruvateformate-lyaseactivatingenzyme	-3,1
S. enteritidis 12538	OKIFFGJM_03565	HollidayjunctionDNAhelicase	-2,7
S. enteritidis 12538	OKIFFGJM_02761	PutativeheatshockproteinYegD	-2,7
S. enteritidis 12538	OKIFFGJM_03490	rRNA(cytosine-C(5)-)-methyltransferaseRsmF	-2,6
S. enteritidis 12538	OKIFFGJM_00832	pathogenicityisland1effectorproteinStpP	-2,5
S. enteritidis 12538	OKIFFGJM_01786	flagellarhook-associatedprotein1	-2,5
S. enteritidis 12538	OKIFFGJM_01829	spermidine/putrescinetransportATP-bindingproteinPotA	-2,3
S. enteritidis 12538	OKIFFGJM_00091	transportprotein	-2,0
S. enteritidis 12538	OKIFFGJM_04324	flagellarmotorswitchproteinFliG	-1,9
S. enteritidis 12538	OKIFFGJM_01585	ssrABactivatedgene	-1,9
S. enteritidis 12538	OKIFFGJM_02937	RtcBprotein	-1,9
S. enteritidis 12538	OKIFFGJM_04347	asparaginesynthetaseAsnA	-1,0
S. enteritidis 12538	OKIFFGJM_03756	ATP-dependentDNAhelicaseRep	-1,0
S. enteritidis 12538	OKIFFGJM_04435	maltoseABCtransporter periplasmicprotein	-1,0
S. enteritidis 12538	OKIFFGJM_01228	inorganicpyrophosphatase	-1,0
S. enteritidis 12538	OKIFFGJM_02719	phosphomannomutase	-0,9
S. enteritidis 12538	OKIFFGJM_00259	MiaBprotein	-0,8
S. enteritidis 12538	OKIFFGJM_00740	L-serinedehydratase2	-0,8
S. enteritidis 12538	OKIFFGJM_02569	adenosinedeaminase	-0,7
S. enteritidis 12538	OKIFFGJM_03249	RibosomalRNAlargesubunitmethyltransferaseN	-0,7
S. enteritidis 12538	OKIFFGJM_01403	glycerol-3-phosphateacyltransferase	-0,7
S. enteritidis 12538	OKIFFGJM_02826	Galactose/methylgalactoside ABCtransport	-0,7

S. enteritidis 12538	OKIFFGJM_04088	RibosomematurationfactorrimM	-0,7
S. enteritidis 12538	OKIFFGJM_03591	purinebindingchemotaxisprotein	-0,7
S. enteritidis 12538	OKIFFGJM_03616	invasionresponse-regulator	-0,7
S. enteritidis 12538	OKIFFGJM_01118	typeIrestrictionendonucleaseStySJIsubunitS	-0,6
S. enteritidis 12538	OKIFFGJM_04683	methionyl-tRNAformyltransferase	-0,6
S. enteritidis 12538	OKIFFGJM_00027	thiaminebiosynthesisproteinThiI	-0,6
S. enteritidis 12538	OKIFFGJM_01993	aminotransferase	-0,6
S. enteritidis 12538	OKIFFGJM_01176	argininedeiminase	-0,6
S. enteritidis 12538	OKIFFGJM_02289	ATP-dependenthelicaseHepA	-0,6
S. enteritidis 12538	OKIFFGJM_00429	ATP-dependentRNAhelicaseDeaD	-0,6
S. enteritidis 12538	OKIFFGJM_03207	phosphoribosylformylglycinamidinesynthase	-0,6
S. enteritidis 12538	OKIFFGJM_02841	1-phosphofructokinase	-0,6
S. enteritidis 12538	OKIFFGJM_01392	replicativeDNAhelicase	-0,6
S. enteritidis 12538	OKIFFGJM_04729	ATP-dependentRNAhelicaseSrmB	-0,6
S. enteritidis 12538	OKIFFGJM_03468	L-serineammonia-lyase	-0,6
S. enteritidis 12538	OKIFFGJM_00020	NutilizationsubstanceproteinB	-0,6
S. enteritidis 12538	OKIFFGJM_00329	Cytochromedubiquinoloxidasesubunit1	-0,6
S. enteritidis 12538	OKIFFGJM_03482	transcriptionalregulatorKdgR	-0,6
S. enteritidis 12538	OKIFFGJM_00378	glutamatesynthasesubunitalpha	-0,6
S. enteritidis 12538	OKIFFGJM_01536	ATP-dependentRNAhelicaseHrpA	-0,6
S. enteritidis 12538	OKIFFGJM_03113	LD-transpeptidaseYbiS	-0,6
S. enteritidis 12538	OKIFFGJM_01487	peptideABCtransportersubstrate-bindingprotein	-0,6
S. enteritidis 12538	OKIFFGJM_02728	glucose-1-phosphatecytidylyltransferase	-0,5
S. enteritidis 12538	OKIFFGJM_03426	ATP/GTP-bindingprotein	-0,5
S. enteritidis 12538	OKIFFGJM_03580	arginyl-tRNAsynthetase	-0,5
S. enteritidis 12538	OKIFFGJM_00132	bifunctionalmethylenetetrahydrofolatedehydrogenase/methenyltetrahydrofolatecyclohydrolase	-0,5
S. enteritidis 12538	OKIFFGJM_02024	amidophosphoribosyltransferase	-0,5

S. enteritidis 12538	OKIFFGJM_02031	semialdehydedehydrogenase	-0,5
S. enteritidis 12538	OKIFFGJM_01667	23SrRNA(guanine-N-2-)-methyltransferaserlmL	-0,5
S. enteritidis 12538	OKIFFGJM_02601	superoxidedismutase	-0,5
S. enteritidis 12538	OKIFFGJM_01835	adenylosuccinatelyase	-0,5
S. enteritidis 12538	OKIFFGJM_02730	dTDP-4-dehydrorhamnose3,5-epimerase	-0,5
S. enteritidis 12538	OKIFFGJM_02684	phosphoenolpyruvatesynthase	-0,5
S. enteritidis 12538	OKIFFGJM_04191	asparaginyl-tRNAsynthetase	-0,5
S. enteritidis 12538	OKIFFGJM_01082	peptidechainreleasefactor3	-0,5
S. enteritidis 12538	OKIFFGJM_03255	GTP-bindingproteinEngA	-0,5
S. enteritidis 12538	OKIFFGJM_00620	S-adenosylmethioninesynthase	-0,5
S. enteritidis 12538	OKIFFGJM_01941	ribonucleoside-diphosphatereductase1subunitbeta	-0,5
S. enteritidis 12538	OKIFFGJM_00221	lipoicacidsynthetase	-0,5
S. enteritidis 12538	OKIFFGJM_02987	celldivisionproteinFtsY	-0,4
S. enteritidis 12538	OKIFFGJM_01086	RibosomalRNAsmallsubunitmethyltransferaseC	-0,4
S. enteritidis 12538	OKIFFGJM_02918	phosphoenolpyruvatecarboxykinase	-0,4
S. enteritidis 12538	OKIFFGJM_02205	pantoate--beta-alanineligase	-0,4
S. enteritidis 12538	OKIFFGJM_02143	prolyl-tRNAsynthetase	-0,4
S. enteritidis 12538	OKIFFGJM_01825	spermidine/putrescine-bindingperiplasmicprotein	-0,4
S. enteritidis 12538	OKIFFGJM_02351	glycine-tRNAsynthetasesubunitalpha	-0,4
S. enteritidis 12538	OKIFFGJM_01819	transcription-repaircouplingfactor(TrcF)	-0,4
S. enteritidis 12538	OKIFFGJM_03190	GTP-bindingproteinLepA	-0,4
S. enteritidis 12538	OKIFFGJM_00802	DNAmismatchrepairproteinMutS	-0,4
S. enteritidis 12538	OKIFFGJM_03422	ribose-phosphatepyrophosphokinase	-0,4
S. enteritidis 12538	OKIFFGJM_00509	ADP-heptosesynthase	-0,4
S. enteritidis 12538	OKIFFGJM_01806	PTSsystemglucose-specifictransportersubunitsIIBC	-0,4
S. enteritidis 12538	OKIFFGJM_01496	transcriptionalregulatorTyrR	-0,4

S. enteritidis 12538	OKIFFGJM_00024	1-deoxy-D-xylulose-5-phosphatesynthase	-0,4
S. enteritidis 12538	OKIFFGJM_00563	hydrogenase-2largesubunit	-0,4
S. enteritidis 12538	OKIFFGJM_04490	glutathioneABCtransporterATP-bindingprotein	-0,4
S. enteritidis 12538	OKIFFGJM_00084	hypotheticalproteinco-occurringwithRecR	-0,4
S. enteritidis 12538	OKIFFGJM_04363	Glucosamine--fructose-6-phosphateaminotransferase[isomerizing]	-0,4
S. enteritidis 12538	OKIFFGJM_01248	50sribosomalsubunitproteinL9	-0,4
S. enteritidis 12538	OKIFFGJM_04662	50SribosomalproteinL5	-0,4
S. enteritidis 12538	OKIFFGJM_03227	RNAmethyltransferase	-0,4
S. enteritidis 12538	OKIFFGJM_00965	rodshape-determiningproteinmreB	-0,4
S. enteritidis 12538	OKIFFGJM_02168	elongationfactorTs	-0,4
S. enteritidis 12538	OKIFFGJM_04790	beta-lactamaseTEM	-0,4
S. enteritidis 12538	OKIFFGJM_03576	tRNA (uridine-5-oxyaceticacidmethylester)34synthase	-0,4
S. enteritidis 12538	OKIFFGJM_00047	putativelipoproteinYajGprecursor	-0,4
S. enteritidis 12538	OKIFFGJM_04664	30SribosomalproteinS8	-0,4
S. enteritidis 12538	OKIFFGJM_04734	tRNA/rRNAmethyltransferaseYfiF	-0,4
S. enteritidis 12538	OKIFFGJM_04399	23SrRNApseudouridinesynthaseF	-0,4
S. enteritidis 12538	OKIFFGJM_03131	ATP-dependentRNAhelicaserhlE	-0,4
S. enteritidis 12538	OKIFFGJM_04356	ATPsynthasesubunitdelta	-0,4
S. enteritidis 12538	OKIFFGJM_00007	queuinetRNA-ribosyltransferase;tRNA-guaninetransglycosylase	-0,4
S. enteritidis 12538	OKIFFGJM_00616	RibosomalRNAsmallsubunitmethyltransferaseE	-0,4
S. enteritidis 12538	OKIFFGJM_03029	zinc-protease	-0,4
S. enteritidis 12538	OKIFFGJM_04661	50SribosomalsubunitproteinL24	-0,4
S. enteritidis 12538	OKIFFGJM_00526	cyclic3',5'-adenosinemonophosphatephosphodiesterase	-0,4
S. enteritidis 12538	OKIFFGJM_02253	celldivisionproteinFtsZ	-0,3

S. enteritidis 12538	OKIFFGJM_00275	glutaminyl-tRNAsynthetase	-0,3
S. enteritidis 12538	OKIFFGJM_04534	50SribosomalsubunitproteinL10	-0,3
S. enteritidis 12538	OKIFFGJM_03457	septumsitedeterminingprotein	-0,3
S. enteritidis 12538	OKIFFGJM_01275	adenylosuccinatesynthetase	-0,3
S. enteritidis 12538	OKIFFGJM_01672	3-hydroxydecanoyl-[acyl-carrier-protein]dehydratase	-0,3
S. enteritidis 12538	OKIFFGJM_02695	phenylalanyl-tRNAsynthetasesubunitbeta	-0,3
S. enteritidis 12538	OKIFFGJM_03273	uracilphosphoribosyltransferase	-0,3
S. enteritidis 12538	OKIFFGJM_04537	transcriptionantiterminationprotein	-0,3
S. enteritidis 12538	OKIFFGJM_00912	DNA-bindingproteinStpA	-0,3
S. enteritidis 12538	OKIFFGJM_04362	UDP-N-acetylglucosaminepyrophosphorylase	-0,3
S. enteritidis 12538	OKIFFGJM_00645	ribose5-phosphateisomerase	-0,3
S. enteritidis 12538	OKIFFGJM_04170	cytidylatekinase	-0,3
S. enteritidis 12538	OKIFFGJM_01814	NADHdehydrogenase	-0,3
S. enteritidis 12538	OKIFFGJM_01940	RibonucleotidereductaseofclassIa(aerobic)alphasubunit	-0,3
S. enteritidis 12538	OKIFFGJM_04665	50SribosomalproteinL6	-0,3
S. enteritidis 12538	OKIFFGJM_04536	50SribosomalproteinL11	-0,3
S. enteritidis 12538	OKIFFGJM_03414	2-dehydro-3-deoxyphosphooctonatealdolase	-0,3
S. enteritidis 12538	OKIFFGJM_00087	adenylatekinase	-0,3
S. enteritidis 12538	OKIFFGJM_03759	thioredoxin	-0,3
S. enteritidis 12538	OKIFFGJM_04448	DNApolymeraseIIIsubunitbeta	-0,3
S. enteritidis 12538	OKIFFGJM_03112	hemeABCtransporterATP-bindingprotein	-0,3
S. enteritidis 12538	OKIFFGJM_02791	methionyl-tRNAsynthetase	-0,3
S. enteritidis 12538	OKIFFGJM_04477	elongationfactorG	-0,3
S. enteritidis 12538	OKIFFGJM_04359	ATPsynthasesubunitbeta	-0,3
S. enteritidis 12538	OKIFFGJM_02911	penicillin-bindingprotein1A	-0,3
S. enteritidis 12538	OKIFFGJM_00758	CTPsynthetase	-0,3
S. enteritidis 12538	OKIFFGJM_00049	triggerfactor	-0,3

S. enteritidis 12538	OKIFFGJM_02406	ADP-heptose-LPSheptosyltransferaseII	-0,3
S. enteritidis 12538	OKIFFGJM_02016	histidine-bindingperiplasmicprotein	-0,3
S. enteritidis 12538	OKIFFGJM_01551	alcoholdehydrogenaseclassIII	-0,3
S. enteritidis 12538	OKIFFGJM_04241	FdhEprotein	-0,3
S. enteritidis 12538	OKIFFGJM_04535	50SribosomalproteinL1	-0,2
S. enteritidis 12538	OKIFFGJM_02350	glycine-tRNAsynthetasesubunitbeta	-0,2
S. enteritidis 12538	OKIFFGJM_00669	lysyltRNAsynthetase	-0,2
S. enteritidis 12538	OKIFFGJM_03418	peptidechainreleasefactor1(RF-1)	-0,2
S. enteritidis 12538	OKIFFGJM_00884	alanyl-tRNAsynthetase	-0,2
S. enteritidis 12538	OKIFFGJM_04446	DNAgyrasesubunitB	-0,2
S. enteritidis 12538	OKIFFGJM_04340	D-ribose-binding periplasmicprotein	-0,2
S. enteritidis 12538	OKIFFGJM_04171	30SribosomalproteinS1	-0,2
S. enteritidis 12538	OKIFFGJM_02696	phenylalanyl-tRNA synthetase subunitalpha	-0,2
S. enteritidis 12538	OKIFFGJM_02181	iron-sulfurclusterinsertionproteinErpA	-0,2
S. enteritidis 12538	OKIFFGJM_00236	leucyl-tRNAsynthetase	-0,2
S. enteritidis 12538	OKIFFGJM_02566	fumaraseA	-0,2
S. enteritidis 12538	OKIFFGJM_02183	glutamate-1-semialdehyde2,1-aminomutase	-0,2
S. enteritidis 12538	OKIFFGJM_00959	biotincarboxylase	-0,2
S. enteritidis 12538	OKIFFGJM_00287	phosphoglucomutase	-0,2
S. enteritidis 12538	OKIFFGJM_04531	DNA-directed RNA polymerasesubunitbeta'	-0,2
**A RU vs A constitutive**			
**S. Enteritidis12539**			
**Strain**	**Protein ID/FASTA Head**	**Protein Name**	**Expression**
S. Enteritidis 12539	GMBILEAH_04135	potassium-transportingATPasesubunitB	7,8
S. Enteritidis 12539	GMBILEAH_02595	oxidativestressdefenseprotein	7,2
S. Enteritidis 12539	GMBILEAH_00899	ABCtransportATP-bindingsubunit	7,2
S. Enteritidis 12539	GMBILEAH_04134	potassium-transportingATPaseAchain	6,0
S. Enteritidis 12539	GMBILEAH_03013	enterobactinsynthetasecomponentF	6,0

S. Enteritidis 12539	GMBILEAH_01957	putativemembraneproteinYqjD	5,6
S. Enteritidis 12539	GMBILEAH_00897	bifunctionalcysteinedesulfurase/selenocysteinelyase	5,3
S. Enteritidis 12539	GMBILEAH_00690	PTSsystemmannitol-specifictransportersubunitIIC	5,2
S. Enteritidis 12539	GMBILEAH_03707	branched-chainaminoacidaminotransferase	5,1
S. Enteritidis 12539	GMBILEAH_01490	cysQprotein	5,0
S. Enteritidis 12539	GMBILEAH_02922	thiosulfate-bindingprotein	4,9
S. Enteritidis 12539	GMBILEAH_03023	2,3-dihydro-2,3-dihydroxybenzoatedehydrogenase	4,7
S. Enteritidis 12539	GMBILEAH_04438	putativeflavoproteinYqcA(clusteredwithtRNApseudouridinesynthaseC)	4,7
S. Enteritidis 12539	GMBILEAH_02355	OsmoprotectantABCtransporterbindingproteinYehZ	4,7
S. Enteritidis 12539	GMBILEAH_03021	enterobactinsynthasesubunitE	4,4
S. Enteritidis 12539	GMBILEAH_02816	glycinebetaine-bindingperiplasmicprotein	4,3
S. Enteritidis 12539	GMBILEAH_03682	irontransportperiplasmic-bindingprotein	4,1
S. Enteritidis 12539	GMBILEAH_03010	outermembranereceptorFepA	4,0
S. Enteritidis 12539	GMBILEAH_01574	acidphosphatase	3,9
S. Enteritidis 12539	GMBILEAH_01777	glucosedehydrogenase	3,9
S. Enteritidis 12539	GMBILEAH_00898	cysteinedesulfuraseactivatorcomplexsubunitSufD	3,8
S. Enteritidis 12539	GMBILEAH_03884	MlctranscriptionalrepressorofMalT(thetranscriptionalactivatorofmaltoseregulon)andmanXYZoperon	3,7
S. Enteritidis 12539	GMBILEAH_04339	thiazolesynthase	3,7
S. Enteritidis 12539	GMBILEAH_01191	LeadcadmiumzincandmercurytransportingATPase	3,7
S. Enteritidis 12539	GMBILEAH_00088	trehalose-6-phosphatesynthase	3,4
S. Enteritidis 12539	GMBILEAH_00652	2-hydroxyaciddehydrogenase	3,3
S. Enteritidis 12539	GMBILEAH_01110	glutaredoxin	3,3
S. Enteritidis 12539	GMBILEAH_00614	putativeproteinYhjG	3,2

S. Enteritidis 12539	GMBILEAH_03001	putativeproteinybdG	3,2
S. Enteritidis 12539	GMBILEAH_01646	quinoneoxidoreductase	3,0
S. Enteritidis 12539	GMBILEAH_01718	3-isopropylmalatedehydrogenase	3,0
S. Enteritidis 12539	GMBILEAH_03837	ferredoxin--NADPreductase	3,0
S. Enteritidis 12539	GMBILEAH_00993	proteinyeaG	2,8
S. Enteritidis 12539	GMBILEAH_03348	lyticmureintransglycosylase	2,5
S. Enteritidis 12539	GMBILEAH_03207	PhosphoglycolatephosphatasePGPase'	2,4
S. Enteritidis 12539	GMBILEAH_03953	phosphateABCtransporterperiplasmicsubstrate-bindingproteinPstS	2,0
S. Enteritidis 12539	GMBILEAH_02353	D-lactatedehydrogenase	1,9
S. Enteritidis 12539	GMBILEAH_04109	TolQprotein	1,7
S. Enteritidis 12539	GMBILEAH_04256	CDP-diacylglycerolpyrophosphatase	1,6
S. Enteritidis 12539	GMBILEAH_03708	dihydroxyaciddehydratase	1,5
S. Enteritidis 12539	GMBILEAH_03709	threoninedehydratase	1,4
S. Enteritidis 12539	GMBILEAH_02949	transaldolaseA	1,4
S. Enteritidis 12539	GMBILEAH_00824	outermembranelipoproteinSlyB	1,3
S. Enteritidis 12539	GMBILEAH_03331	OsmoticallyinducibleproteinOsmY	1,3
S. Enteritidis 12539	GMBILEAH_00357	D-alanyl-D-alaninecarboxypeptidasefractionC	1,2
S. Enteritidis 12539	GMBILEAH_00943	6-phosphofructokinase2	1,2
S. Enteritidis 12539	GMBILEAH_01214	acriflavinresistanceproteinA;acriflavinresistanceproteinE	1,2
S. Enteritidis 12539	GMBILEAH_00233	outermembraneproteinA	1,2
S. Enteritidis 12539	GMBILEAH_00374	ABCtransporterperiplasmicbindingprotein	1,2
S. Enteritidis 12539	GMBILEAH_00030	alkyl/aryl-sulfataseBDS1	1,2
S. Enteritidis 12539	GMBILEAH_02908	cysteinesynthaseA	1,2
S. Enteritidis 12539	GMBILEAH_01914	outermembraneproteinTolC	1,1
S. Enteritidis 12539	GMBILEAH_03003	dihydropteridinereductase	1,1
S. Enteritidis 12539	GMBILEAH_01215	acriflavinresistanceproteinB	1,1
S. Enteritidis 12539	GMBILEAH_00313	ATP-dependentClpproteaseATP-bindingsubunit	1,0

S. Enteritidis 12539	GMBILEAH_04246	superoxidedismutase	1,0
S. Enteritidis 12539	GMBILEAH_02449	histidinoldehydrogenase	1,0
S. Enteritidis 12539	GMBILEAH_04124	citratesynthase	1,0
S. Enteritidis 12539	GMBILEAH_03137	ecotin	1,0
S. Enteritidis 12539	GMBILEAH_01886	biopolymertransportproteinExbB	1,0
S. Enteritidis 12539	GMBILEAH_02450	ATPphosphoribosyltransferase	0,9
S. Enteritidis 12539	GMBILEAH_03172	lipopolysaccharidemodificationprotein	0,9
S. Enteritidis 12539	GMBILEAH_02236	aconitatehydratase1	0,8
S. Enteritidis 12539	GMBILEAH_02444	cyclaseHisF	0,8
S. Enteritidis 12539	GMBILEAH_00767	NicotinamidasefamilyproteinYcaC	0,8
S. Enteritidis 12539	GMBILEAH_03208	helix-turn-helixdomain-containingprotein	0,8
S. Enteritidis 12539	GMBILEAH_03974	sugarphosphatephosphatase	0,8
S. Enteritidis 12539	GMBILEAH_01037	isocitratedehydrogenase	0,8
S. Enteritidis 12539	GMBILEAH_03798	phosphoenolpyruvatecarboxylase	0,8
S. Enteritidis 12539	GMBILEAH_03142	outermembraneproteinC	0,7
S. Enteritidis 12539	GMBILEAH_02033	putativeABCtransporterauxiliarycomponentYrbC	0,7
S. Enteritidis 12539	GMBILEAH_02694	Decarboxylasefamilyprotein	0,7
S. Enteritidis 12539	GMBILEAH_03107	N-acetylglucosamine-6-phosphatedeacetylase	0,7
S. Enteritidis 12539	GMBILEAH_02071	serineprotease	0,7
S. Enteritidis 12539	GMBILEAH_02070	cytochromedubiquinoloxidasesubunitIII	0,7
S. Enteritidis 12539	GMBILEAH_01437	cytosolaminopeptidase	0,7
S. Enteritidis 12539	GMBILEAH_04462	phosphoglyceromutase	0,7
S. Enteritidis 12539	GMBILEAH_03907	3-hydroxyaciddehydrogenase	0,7
S. Enteritidis 12539	GMBILEAH_01891	alcoholdehydrogenase	0,7
S. Enteritidis 12539	GMBILEAH_00508	osmolarityresponseregulator	0,7
S. Enteritidis 12539	GMBILEAH_04146	malatedehydrogenase	0,7
S. Enteritidis 12539	GMBILEAH_01484	putativeproteinYtfN	0,7
S. Enteritidis 12539	GMBILEAH_00285	phosphoserineaminotransferase	0,7
S. Enteritidis 12539	GMBILEAH_00245	aminopeptidaseN	0,6

S. Enteritidis 12539	GMBILEAH_03367	aspartokinaseI/homoserine dehydrogenaseI	0,6
S. Enteritidis 12539	GMBILEAH_02605	aminomethyltransferase	0,6
S. Enteritidis 12539	GMBILEAH_01698	organicsolventtoleranceprotein	0,6
S. Enteritidis 12539	GMBILEAH_01823	2,3,4,5-tetrahydropyridine-2,6-carboxylateN-succinyltransferase	0,6
S. Enteritidis 12539	GMBILEAH_02703	chorismatemutase-P/prephenatedehydratase	0,6
S. Enteritidis 12539	GMBILEAH_00539	glycogenphosphorylase	0,6
S. Enteritidis 12539	GMBILEAH_00974	nitroreductase	0,6
S. Enteritidis 12539	GMBILEAH_02862	long-chainfattyacidtransportprotein	0,6
S. Enteritidis 12539	GMBILEAH_03620	lipoproteinNlpD	0,6
S. Enteritidis 12539	GMBILEAH_03783	prolinedipeptidase	0,6
S. Enteritidis 12539	GMBILEAH_02698	proteindisaggregationchaperone	0,6
S. Enteritidis 12539	GMBILEAH_03094	phosphatestarvation-inducibleprotein,ATP-binding	0,6
S. Enteritidis 12539	GMBILEAH_03072	rarelipoproteinB	0,5
S. Enteritidis 12539	GMBILEAH_01125	glucanbiosynthesisproteinG	0,5
S. Enteritidis 12539	GMBILEAH_01765	bifunctionalaconitatehydratase2/2-methylisocitratedehydratase	0,5
S. Enteritidis 12539	GMBILEAH_02032	sulfatetransporter	0,5
S. Enteritidis 12539	GMBILEAH_02948	NADP-dependentmalatedehydrogenase	0,5
S. Enteritidis 12539	GMBILEAH_00110	deaminase	0,5
S. Enteritidis 12539	GMBILEAH_00381	hydrolase(HADsuperfamily);putativehydrolase	0,5
S. Enteritidis 12539	GMBILEAH_00893	majoroutermembranelipoprotein	0,5
S. Enteritidis 12539	GMBILEAH_03840	triosephosphateisomerase	0,5
S. Enteritidis 12539	GMBILEAH_03863	PutativeGTP-bindingproteinYdgA	0,4
S. Enteritidis 12539	GMBILEAH_00598	oligopeptidaseA	0,4
S. Enteritidis 12539	GMBILEAH_01531	FtsHproteaseregulatorHflC	0,4
S. Enteritidis 12539	GMBILEAH_01368	Glutamate5-kinase	0,4
S. Enteritidis 12539	GMBILEAH_03339	phosphopentomutase	0,4
S. Enteritidis 12539	GMBILEAH_02551	putativeFe(2+)-traffickingproteinYggX	0,4

S. Enteritidis 12539	GMBILEAH_03369	threoninesynthase	0,4
S. Enteritidis 12539	GMBILEAH_01185	Thioredoxindomain-containingproteinEC-YbbN	0,4
S. Enteritidis 12539	GMBILEAH_03554	glutamatedehydrogenase	0,4
S. Enteritidis 12539	GMBILEAH_01479	fructose-1,6-bisphosphatase	0,4
S. Enteritidis 12539	GMBILEAH_03807	transaldolase	0,4
S. Enteritidis 12539	GMBILEAH_03401	RNApolymerasesigma-Efactor(sigma-24)	0,4
S. Enteritidis 12539	GMBILEAH_01195	bifunctionalUDP-sugarhydrolase/5'-nucleotidase	0,4
S. Enteritidis 12539	GMBILEAH_01238	Lonprotease	0,4
S. Enteritidis 12539	GMBILEAH_04301	Glucose-6-phosphateisomerase	0,4
S. Enteritidis 12539	GMBILEAH_03195	NADHdehydrogenasesubunitI	0,4
S. Enteritidis 12539	GMBILEAH_04119	2-oxoglutarate dehydrogenase E1component	0,4
S. Enteritidis 12539	GMBILEAH_00844	riboflavinsynthasealphachain	0,4
S. Enteritidis 12539	GMBILEAH_02523	H-NShistonefamilyprotein	0,4
S. Enteritidis 12539	GMBILEAH_01331	typeIIIrestriction-modificationsystemStyLTIenzymemod	0,3
S. Enteritidis 12539	GMBILEAH_02591	phosphoglyceratekinase	0,3
S. Enteritidis 12539	GMBILEAH_02905	DNAligase	0,3
S. Enteritidis 12539	GMBILEAH_01373	aminoacyl-histidinedipeptidase	0,3
S. Enteritidis 12539	GMBILEAH_02567	glutathionesynthetase	0,3
S. Enteritidis 12539	GMBILEAH_02804	DNA-bindingproteinStpA	0,3
S. Enteritidis 12539	GMBILEAH_00043	KHG/KDPGaldolase	0,3
S. Enteritidis 12539	GMBILEAH_03452	peptidaseB	0,3
S. Enteritidis 12539	GMBILEAH_00528	DNA-bindingtranscriptionalrepressorGlpR	0,3
S. Enteritidis 12539	GMBILEAH_03372	transaldolaseB	0,3
S. Enteritidis 12539	GMBILEAH_00472	cyclicAMPreceptorprotein,catabolitegeneactivator	0,3
S. Enteritidis 12539	GMBILEAH_00258	nicotinatephosphoribosyltransferase	0,2
S. Enteritidis 12539	GMBILEAH_04116	succinyl-CoAsynthetasesubunitalpha	0,2
S. Enteritidis 12539	GMBILEAH_00045	glucose6-phosphatedehydrogenase	0,2

S. Enteritidis 12539	GMBILEAH_03445	L-cysteinedesulfurase	0,2
S. Enteritidis 12539	GMBILEAH_01798	penicillin-bindingprotein1b	0,2
S. Enteritidis 12539	GMBILEAH_03464	nucleosidediphosphatekinase(ndk)	0,2
S. Enteritidis 12539	GMBILEAH_01239	ATP-dependentclpproteaseATP-bindingsubunitClpX	0,2
S. Enteritidis 12539	GMBILEAH_03122	phosphoglucomutase	0,2
S. Enteritidis 12539	GMBILEAH_01695	dimethyladenosinetransferase	-5,5
S. Enteritidis 12539	GMBILEAH_03933	asparaginesynthetaseAsnA	-5,3
S. Enteritidis 12539	GMBILEAH_04071	plasmidpartitionproteinB	-5,2
S. Enteritidis 12539	GMBILEAH_03965	InnermembraneproteintranslocasecomponentYidClongform	-4,9
S. Enteritidis 12539	GMBILEAH_02398	Uridinekinase[C1]	-4,3
S. Enteritidis 12539	GMBILEAH_00405	ATP-dependentRNAhelicaserhlE	-4,2
S. Enteritidis 12539	GMBILEAH_00745	guanylatekinase	-4,2
S. Enteritidis 12539	GMBILEAH_03859	Cnuprotein	-4,1
S. Enteritidis 12539	GMBILEAH_02156	UniversalstressproteinF	-4,0
S. Enteritidis 12539	GMBILEAH_03770	ubiquinone/menaquinonebiosynthesis methyltransferaseUbiE	-4,0
S. Enteritidis 12539	GMBILEAH_01099	flagellarhookformationproteinFlgD	-3,9
S. Enteritidis 12539	GMBILEAH_00378	molybdopterinbiosynthesisMoeBprotein	-3,8
S. Enteritidis 12539	GMBILEAH_03777	TranscriptionantiterminationproteinnusG	-3,6
S. Enteritidis 12539	GMBILEAH_03465	RibosomalRNAlargesubunitmethyltransferaseN	-3,6
S. Enteritidis 12539	GMBILEAH_02431	paratosesynthase	-3,6
S. Enteritidis 12539	GMBILEAH_03967	chromosomalreplicationinitiationprotein	-3,5
S. Enteritidis 12539	GMBILEAH_03964	tRNAmodificationGTPaseTrmE	-3,5
S. Enteritidis 12539	GMBILEAH_01841	DNApolymeraseIIIsubunitalpha	-3,4
S. Enteritidis 12539	GMBILEAH_03543	Fattyacidmetabolismregulatorprotein	-3,2
S. Enteritidis 12539	GMBILEAH_04332	uroporphyrinogendecarboxylase	-3,1
S. Enteritidis 12539	GMBILEAH_04432	23SrRNA(uracil-5-)-methyltransferaseRumA	-2,7

S. Enteritidis 12539	GMBILEAH_01930	putativeproteinygiFORFXE	-2,6
S. Enteritidis 12539	GMBILEAH_04396	putativeribosomematurationfactor	-2,3
S. Enteritidis 12539	GMBILEAH_01526	putativetRNA/rRNAmethyltransferase	-2,2
S. Enteritidis 12539	GMBILEAH_03061	putativelipoateregulatoryproteinYbeD	-1,9
S. Enteritidis 12539	GMBILEAH_00716	ADP-heptose-LPSheptosyltransferaseII	-1,6
S. Enteritidis 12539	GMBILEAH_02002	ATP-dependentRNAhelicaseDeaD	-1,6
S. Enteritidis 12539	GMBILEAH_02692	L-serinedehydratase2	-1,6
S. Enteritidis 12539	GMBILEAH_00432	biotinsynthetase	-1,5
S. Enteritidis 12539	GMBILEAH_00067	ProteinyecM	-1,4
S. Enteritidis 12539	GMBILEAH_03533	septumsitedeterminingprotein	-1,0
S. Enteritidis 12539	GMBILEAH_02020	RNAbindingprotein	-1,0
S. Enteritidis 12539	GMBILEAH_04361	50SribosomalsubunitproteinL2	-1,0
S. Enteritidis 12539	GMBILEAH_04366	50SribosomalsubunitproteinL29	-1,0
S. Enteritidis 12539	GMBILEAH_03442	extragenicsuppressorproteinSuhB	-1,0
S. Enteritidis 12539	GMBILEAH_00386	hemeABCtransporterATP-bindingprotein	-1,0
S. Enteritidis 12539	GMBILEAH_04362	30SribosomalsubunitproteinS19	-0,9
S. Enteritidis 12539	GMBILEAH_02755	AbortivephageinfectionproteinAIPR	-0,9
S. Enteritidis 12539	GMBILEAH_02067	30SribosomalsubunitproteinS9	-0,9
S. Enteritidis 12539	GMBILEAH_01645	replicativeDNAhelicase	-0,9
S. Enteritidis 12539	GMBILEAH_04161	rodshape-determiningproteinmreB	-0,8
S. Enteritidis 12539	GMBILEAH_04369	50SribosomalsubunitproteinL24	-0,8
S. Enteritidis 12539	GMBILEAH_04293	maltoseABCtransporterperiplasmicprotein	-0,7
S. Enteritidis 12539	GMBILEAH_00453	30SribosomalsubunitproteinS7	-0,7
S. Enteritidis 12539	GMBILEAH_04365	50SribosomalproteinL16	-0,7
S. Enteritidis 12539	GMBILEAH_02709	50SribosomalsubunitproteinL19	-0,7
S. Enteritidis 12539	GMBILEAH_04323	phosphoribosylaminoimidazolecarboxamideformyltransferaseandIMP cyclohydrolase	-0,7
S. Enteritidis 12539	GMBILEAH_01503	30sribosomalsubunitproteinS18	-0,7

S. Enteritidis 12539	GMBILEAH_02331	Galactose/methylgalactoside ABCtransport	-0,7
S. Enteritidis 12539	GMBILEAH_03058	lipoicacidsynthetase	-0,7
S. Enteritidis 12539	GMBILEAH_03574	peptidechainreleasefactor1(RF-1)	-0,6
S. Enteritidis 12539	GMBILEAH_04352	elongationfactorTu	-0,6
S. Enteritidis 12539	GMBILEAH_02146	ATP-dependentRNAhelicaseHrpA	-0,6
S. Enteritidis 12539	GMBILEAH_01939	RNApolymerasesigma-70factor	-0,6
S. Enteritidis 12539	GMBILEAH_04370	50SribosomalproteinL5	-0,6
S. Enteritidis 12539	GMBILEAH_04359	50SribosomalsubunitproteinL4	-0,6
S. Enteritidis 12539	GMBILEAH_01152	cysteinyl-tRNAsynthetase	-0,5
S. Enteritidis 12539	GMBILEAH_04384	50SribosomalsubunitproteinL17	-0,5
S. Enteritidis 12539	GMBILEAH_01738	celldivisionproteinFtsZ	-0,5
S. Enteritidis 12539	GMBILEAH_03152	ribonucleoside-diphosphatereductase1subunitbeta	-0,5
S. Enteritidis 12539	GMBILEAH_00840	purinenucleotidesynthesisrepressor	-0,5
S. Enteritidis 12539	GMBILEAH_04358	50SribosomalproteinL3	-0,5
S. Enteritidis 12539	GMBILEAH_02010	ribosomematurationproteinRimP	-0,5
S. Enteritidis 12539	GMBILEAH_01728	UDP-N-acetylmuramoylalanyl-D-glutamate--2,6-diaminopimligase	-0,5
S. Enteritidis 12539	GMBILEAH_01081	malonylCoA-ACPtransacylase	-0,5
S. Enteritidis 12539	GMBILEAH_00241	23SrRNA(guanine-N-2-)-methyltransferaserlmL	-0,5
S. Enteritidis 12539	GMBILEAH_03719	transcriptionterminationfactorRho	-0,5
S. Enteritidis 12539	GMBILEAH_03751	DNAhelicaseII	-0,4
S. Enteritidis 12539	GMBILEAH_04382	30SribosomalproteinS4	-0,4
S. Enteritidis 12539	GMBILEAH_01505	30sribosomalproteinS6	-0,4
S. Enteritidis 12539	GMBILEAH_04375	30SribosomalproteinS5	-0,4
S. Enteritidis 12539	GMBILEAH_00733	50SribosomalproteinL28	-0,4
S. Enteritidis 12539	GMBILEAH_00461	FKBP-typepeptidyl-prolylcis-transisomerase	-0,4
S. Enteritidis 12539	GMBILEAH_02677	CsdA-bindingactivator	-0,4
S. Enteritidis 12539	GMBILEAH_03393	tRNA/rRNAmethyltransferaseYfiF	-0,4

S. Enteritidis 12539	GMBILEAH_03948	UDP-N-acetylglucosaminepyrophosphorylase	-0,4
S. Enteritidis 12539	GMBILEAH_01270	NutilizationsubstanceproteinB	-0,4
S. Enteritidis 12539	GMBILEAH_01723	fructoserepressor	-0,4
S. Enteritidis 12539	GMBILEAH_04363	50SribosomalproteinL22	-0,4
S. Enteritidis 12539	GMBILEAH_00281	30SribosomalproteinS1	-0,4
S. Enteritidis 12539	GMBILEAH_01072	PTSsystemglucose-specifictransportersubunitsIIBC	-0,4
S. Enteritidis 12539	GMBILEAH_00452	elongationfactorG	-0,3
S. Enteritidis 12539	GMBILEAH_02225	exoribonucleaseII	-0,3
S. Enteritidis 12539	GMBILEAH_04380	30SribosomalproteinS13	-0,3
S. Enteritidis 12539	GMBILEAH_02009	Lfactor	-0,3
S. Enteritidis 12539	GMBILEAH_03211	phosphateacetyltransferase	-0,3
S. Enteritidis 12539	GMBILEAH_03570	ribose-phosphatepyrophosphokinase	-0,3
S. Enteritidis 12539	GMBILEAH_00575	celldivisionproteinFtsY	-0,3
S. Enteritidis 12539	GMBILEAH_01241	triggerfactor	-0,3
S. Enteritidis 12539	GMBILEAH_03151	RibonucleotidereductaseofclassIa(aerobic)alphasubunit	-0,3
S. Enteritidis 12539	GMBILEAH_03945	ATPsynthasesubunitbeta	-0,3
S. Enteritidis 12539	GMBILEAH_00105	invasionresponse-regulator	-0,3
S. Enteritidis 12539	GMBILEAH_04347	50SribosomalsubunitproteinL10	-0,3
S. Enteritidis 12539	GMBILEAH_04373	50SribosomalproteinL6	-0,3
S. Enteritidis 12539	GMBILEAH_04377	50SribosomalproteinL15	-0,3
S. Enteritidis 12539	GMBILEAH_02295	nucleoid-asociatedprotein	-0,3
S. Enteritidis 12539	GMBILEAH_01439	valyl-tRNAsynthetase	-0,3
S. Enteritidis 12539	GMBILEAH_03970	DNAgyrasesubunitB	-0,3
S. Enteritidis 12539	GMBILEAH_04383	DNA-directedRNApolymerasesubunitalpha	-0,3
S. Enteritidis 12539	GMBILEAH_00937	threonyl-tRNAsynthetase	-0,3
S. Enteritidis 12539	GMBILEAH_00933	phenylalanyl-tRNAsynthetasesubunitalpha	-0,3
S. Enteritidis 12539	GMBILEAH_00660	glycine-tRNAsynthetasesubunitbeta	-0,3

S. Enteritidis 12539	GMBILEAH_02008	proteinchaininitiationfactor2	-0,3
S. Enteritidis 12539	GMBILEAH_02713	signalrecognitionparticleprotein	-0,3
S. Enteritidis 12539	GMBILEAH_01502	50sribosomalsubunitproteinL9	-0,3
S. Enteritidis 12539	GMBILEAH_01202	adenylatekinase	-0,3
S. Enteritidis 12539	GMBILEAH_03592	CTPsynthetase	-0,3
S. Enteritidis 12539	GMBILEAH_03073	leucyl-tRNAsynthetase	-0,2
S. Enteritidis 12539	GMBILEAH_01080	3-oxoacyl-ACPreductase	-0,2
S. Enteritidis 12539	GMBILEAH_03943	ATPsynthasesubunitalpha	-0,2
S. Enteritidis 12539	GMBILEAH_00064	tRNA(uridine-5-oxyaceticacidmethylester)34synthase	-0,2
S. Enteritidis 12539	GMBILEAH_00068	arginyl-tRNAsynthetase	-0,2
S. Enteritidis 12539	GMBILEAH_01852	prolyl-tRNAsynthetase	-0,2
S. Enteritidis 12539	GMBILEAH_02711	RibosomematurationfactorrimM	-0,2
S. Enteritidis 12539	GMBILEAH_04345	DNA-directedRNApolymerasesubunitbeta	-0,2
S. Enteritidis 12539	GMBILEAH_00387	LD-transpeptidaseYbiS	-0,2
**A RU vs A constitutive**			
**S. typhimurium 12468**			
**Strain**	**Protein ID/FASTA Head**	**Protein Name**	**Expression**
S. typhimurium 12468	PMJHGEPJ_02881	peptidaseB	0,5
S. typhimurium 12468	PMJHGEPJ_02392	acriflavinresistanceproteinA	0,8
S. typhimurium 12468	PMJHGEPJ_00174	adenylosuccinatelyase	-0,5
**A RU vs A constitutive**			
**S. typhimurium 12472**			
**Strain**	**Protein ID/FASTA Head**	**Protein Name**	**Expression**
S. typhimurium 12472	HCMLBOHL_01183	enterobactinsynthetasecomponentF	6,8
S. typhimurium 12472	HCMLBOHL_01190	isochorismatesynthaseEntC	6,0
S. typhimurium 12472	HCMLBOHL_01180	outermembranereceptorFepA	6,0
S. typhimurium 12472	HCMLBOHL_01193	2,3-dihydro-2,3-dihydroxybenzoatedehydrogenase	5,4
S. typhimurium 12472	HCMLBOHL_01191	enterobactinsynthasesubunitE	4,2

S. typhimurium 12472	HCMLBOHL_00543	glycinebetaine-bindingperiplasmicprotein	3,9
S. typhimurium 12472	HCMLBOHL_00492	irontransportperiplasmic-bindingprotein	3,7
S. typhimurium 12472	HCMLBOHL_01189	ferrienterobactin-bindingperiplasmicprotein	3,3
S. typhimurium 12472	HCMLBOHL_01762	bifunctionalcysteinedesulfurase/selenocysteinelyase	2,8
S. typhimurium 12472	HCMLBOHL_01610	Succinate-semialdehydedehydrogenase[NAD]	2,7
S. typhimurium 12472	HCMLBOHL_01192	isochorismatase	2,6
S. typhimurium 12472	HCMLBOHL_02919	Iron-sulfurclusterregulatorIscR	1,7
S. typhimurium 12472	HCMLBOHL_00920	acidphosphatase	1,6
S. typhimurium 12472	HCMLBOHL_03613	CDP-diacylglycerolpyrophosphatase	1,3
S. typhimurium 12472	HCMLBOHL_00195	biopolymertransportproteinExbB	1,2
S. typhimurium 12472	HCMLBOHL_04449	phosphoribosylaminoimidazolecarboxamideformyltransferaseandIMPcyclohydrolase	1,2
S. typhimurium 12472	HCMLBOHL_02178	thiamine-bindingperiplasmicprotein	1,2
S. typhimurium 12472	HCMLBOHL_00540	multidrugresistancesecretionprotein	1,1
S. typhimurium 12472	HCMLBOHL_01614	MultipleantibioticresistanceproteinMarR	1,1
S. typhimurium 12472	HCMLBOHL_02954	inosine5'-monophosphatedehydrogenase	1,1
S. typhimurium 12472	HCMLBOHL_02955	GMPsynthase	0,9
S. typhimurium 12472	HCMLBOHL_03604	superoxidedismutase	0,9
S. typhimurium 12472	HCMLBOHL_01173	dihydropteridinereductase	0,8
S. typhimurium 12472	HCMLBOHL_00764	cytosolaminopeptidase	0,8
S. typhimurium 12472	HCMLBOHL_00297	aminomethyltransferase	0,8
S. typhimurium 12472	HCMLBOHL_02898	phosphoribosylformylglycinamidinesynthase	0,7
S. typhimurium 12472	HCMLBOHL_03667	phosphoenolpyruvatecarboxylase	0,7
S. typhimurium 12472	HCMLBOHL_01082	acriflavinresistanceproteinB	0,7

S. typhimurium 12472	HCMLBOHL_01475	transcriptionalregulatorPhoP,regulatorofvirulencedeterminants	0,6
S. typhimurium 12472	HCMLBOHL_02095	ferrichromeoutermembranetransporter	0,6
S. typhimurium 12472	HCMLBOHL_02927	peptidaseB	0,6
S. typhimurium 12472	HCMLBOHL_01083	acriflavinresistanceproteinA	0,6
S. typhimurium 12472	HCMLBOHL_02801	hydrolase(HADsuperfamily)	0,6
S. typhimurium 12472	HCMLBOHL_01689	outermembranelipoproteinSlyB	0,5
S. typhimurium 12472	HCMLBOHL_00167	outermembraneproteinTolC	0,5
S. typhimurium 12472	HCMLBOHL_04359	malatedehydrogenase	0,5
S. typhimurium 12472	HCMLBOHL_01482	isocitratedehydrogenase	0,5
S. typhimurium 12472	HCMLBOHL_02939	nucleosidediphosphatekinase(ndk)	0,5
S. typhimurium 12472	HCMLBOHL_00908	GroELprotein	0,5
S. typhimurium 12472	HCMLBOHL_01059	Lonprotease	0,5
S. typhimurium 12472	HCMLBOHL_01476	adenylosuccinatelyase	0,4
S. typhimurium 12472	HCMLBOHL_00630	DnaKprotein	0,4
S. typhimurium 12472	HCMLBOHL_02128	bifunctionalaconitatehydratase2/2-methylisocitratedehydratase	0,4
S. typhimurium 12472	HCMLBOHL_03857	KHG/KDPGaldolase	0,4
S. typhimurium 12472	HCMLBOHL_01537	nitroreductase	0,4
S. typhimurium 12472	HCMLBOHL_01316	outermembraneproteinA	0,3
S. typhimurium 12472	HCMLBOHL_01006	maltose/maltodextrintransportATP-bindingprotein	-3,7
S. typhimurium 12472	HCMLBOHL_04323	maltoseABCtransporterperiplasmicprotein	-1,2
S. typhimurium 12472	HCMLBOHL_02625	maltodextrinphosphorylase	-1,1
S. typhimurium 12472	HCMLBOHL_04444	nitritereductaselargesubunit	-1,1
S. typhimurium 12472	HCMLBOHL_04235	asparaginesynthetaseAsnA	-1,0
S. typhimurium 12472	HCMLBOHL_01786	phosphoenolpyruvatesynthase	-0,9
S. typhimurium 12472	HCMLBOHL_00727	isoaspartyldipeptidase	-0,9
S. typhimurium 12472	HCMLBOHL_00073	ATP-dependentRNAhelicaseDeaD	-0,8
S. typhimurium 12472	HCMLBOHL_02531	Galactose/methylgalactosideABCtransport	-0,8
S. typhimurium 12472	HCMLBOHL_03131	coldshockprotein	-0,8
S. typhimurium 12472	HCMLBOHL_03524	ketol-acidreductoisomerase	-0,7

S. typhimurium 12472	HCMLBOHL_00383	L-serinedehydratase2	-0,6
S. typhimurium 12472	HCMLBOHL_04015	formateacetyltransferase1	-0,6
S. typhimurium 12472	HCMLBOHL_00207	hydrogenase-2largesubunit	-0,6
S. typhimurium 12472	HCMLBOHL_01033	thiaminebiosynthesisproteinThiI	-0,5
S. typhimurium 12472	HCMLBOHL_02547	ptssystem,fructose-specificIIA/FPRcomponent	-0,5
S. typhimurium 12472	HCMLBOHL_01266	MiaBprotein	-0,5
S. typhimurium 12472	HCMLBOHL_03634	glycerolkinase	-0,5
S. typhimurium 12472	HCMLBOHL_02868	UDP-glucose4-epimerase	-0,5
S. typhimurium 12472	HCMLBOHL_00642	globalresponseregulator	-0,5
S. typhimurium 12472	HCMLBOHL_04250	UDP-N-acetylglucosaminepyrophosphorylase	-0,4
S. typhimurium 12472	HCMLBOHL_04023	30SribosomalproteinS1	-0,2
S. typhimurium 12472	HCMLBOHL_02043	prolyl-tRNAsynthetase	-0,2
S. typhimurium 12472	HCMLBOHL_00071	Polyribonucleotidenucleotidyltransferase	-0,2
**M consitutitve vs A consitutive**			
**S. Enteritidis12539**			
**Strain**	**Protein ID/FASTA Head**	**Protein Name**	**Expression**
S. Enteritidis 12539	GMBILEAH_02595	oxidativestressdefenseprotein	6,2
S. Enteritidis 12539	GMBILEAH_02310	elongationfactor	5,7
S. Enteritidis 12539	GMBILEAH_03066	LSUmethyltransferaseRlmH	4,9
S. Enteritidis 12539	GMBILEAH_00284	3-phosphoshikimate1-carboxyvinyltransferase	4,6
S. Enteritidis 12539	GMBILEAH_00285	phosphoserineaminotransferase	4,6
S. Enteritidis 12539	GMBILEAH_01985	rRNAsmallsubunitmethyltransferaseI	4,4
S. Enteritidis 12539	GMBILEAH_01659	maltoseoperonperiplasmicprotein	3,9
S. Enteritidis 12539	GMBILEAH_01255	4-methyl-5(b-hydroxyethyl)-thiazolemonophosphatebiosynthesis(ThiJ)protein	3,7
S. Enteritidis 12539	GMBILEAH_03789	tRNA(uracil-5)-methyltransferase	3,0
S. Enteritidis 12539	GMBILEAH_00691	mannitol-1-phosphate5-dehydrogenase	2,9
S. Enteritidis 12539	GMBILEAH_01932	tRNAnucleotidyltransferase	2,2

S. Enteritidis 12539	GMBILEAH_03742	CyaYprotein	0,9
S. Enteritidis 12539	GMBILEAH_01723	fructoserepressor	0,5
S. Enteritidis 12539	GMBILEAH_04464	galactokinase	0,5
S. Enteritidis 12539	GMBILEAH_03398	ATP-dependentRNAhelicaseSrmB	0,4
S. Enteritidis 12539	GMBILEAH_01253	putativenucleotide-bindingprotein	0,4
S. Enteritidis 12539	GMBILEAH_01701	ATP-dependenthelicaseHepA	0,4
S. Enteritidis 12539	GMBILEAH_02295	nucleoid-asociatedprotein	0,3
S. Enteritidis 12539	GMBILEAH_03372	transaldolaseB	0,3
S. Enteritidis 12539	GMBILEAH_02834	recombinaseA	0,3
S. Enteritidis 12539	GMBILEAH_00387	LD-transpeptidaseYbiS	0,2
S. Enteritidis 12539	GMBILEAH_00767	NicotinamidasefamilyproteinYcaC	-3,8
S. Enteritidis 12539	GMBILEAH_01090	ribonucleaseE	-0,5
S. Enteritidis 12539	GMBILEAH_03378	DnaJprotein	-0,4
S. Enteritidis 12539	GMBILEAH_03377	DnaKprotein	-0,4
S. Enteritidis 12539	GMBILEAH_01564	GroELprotein	-0,3
S. Enteritidis 12539	GMBILEAH_02911	ptssystem,glucose-specificIIAcomponent	-0,2
**M consitutitve vs A consitutive**			
**S. typhimurium 12468**			
**Strain**	**Protein ID/FASTA Head**	**Protein Name**	**Expression**
S. typhimurium 12468	PMJHGEPJ_04331	heatshockprotein	7,5
S. typhimurium 12468	PMJHGEPJ_03747	3-phosphoshikimate1-carboxyvinyltransferase	7,3
S. typhimurium 12468	PMJHGEPJ_03442	DNAprotectionduringstarvationprotein	7,2
S. typhimurium 12468	PMJHGEPJ_00688	oligopeptideABCtransportersubstrate-bindingprotein	6,4
S. typhimurium 12468	PMJHGEPJ_02302	OsmoprotectantABCtransporterbinding proteinYehZ	5,9
S. typhimurium 12468	PMJHGEPJ_03773	Isochorismatasefamilyprotein	5,7
S. typhimurium 12468	PMJHGEPJ_03581	high-affinityzinctransporterperiplasmicprotein	5,2

S. typhimurium 12468	PMJHGEPJ_02580	ABCtransporterperiplasmicbindingprotein	4,8
S. typhimurium 12468	PMJHGEPJ_01907	fumaratereductasecomplexsubunitC;membraneanchorpolypeptide	4,5
S. typhimurium 12468	PMJHGEPJ_03407	glutathiones-transferasefamilyprotein	4,2
S. typhimurium 12468	PMJHGEPJ_03748	phosphoserineaminotransferase	4,2
S. typhimurium 12468	PMJHGEPJ_00566	proteinydcJ	4,1
S. typhimurium 12468	PMJHGEPJ_02926	signalpeptidaseI	3,9
S. typhimurium 12468	PMJHGEPJ_00450	dipeptidylcarboxypeptidaseII	2,7
S. typhimurium 12468	PMJHGEPJ_01686	OsmoticallyinducibleproteinOsmY	2,0
S. typhimurium 12468	PMJHGEPJ_04426	bacterioferritin	1,7
S. typhimurium 12468	PMJHGEPJ_01734	isoaspartyldipeptidase	1,3
S. typhimurium 12468	PMJHGEPJ_00065	trifunctionaltranscriptionalregulator/prolinedehydrogenase/pyrroline-5-carboxylatedehydrogenase	1,3
S. typhimurium 12468	PMJHGEPJ_01335	glycerol-3-phosphate-bindingperiplasmicprotein	1,1
S. typhimurium 12468	PMJHGEPJ_03939	ChromosomesegregationATPase	1,0
S. typhimurium 12468	PMJHGEPJ_03192	50SribosomalproteinL21	0,8
S. typhimurium 12468	PMJHGEPJ_04289	citratesynthase	0,6
S. typhimurium 12468	PMJHGEPJ_00389	tyrosyl-tRNAsynthetase	0,4
S. typhimurium 12468	PMJHGEPJ_03014	3-isopropylmalatedehydratase	-5,3
S. typhimurium 12468	PMJHGEPJ_04311	thiazolesynthase	-4,2
S. typhimurium 12468	PMJHGEPJ_02282	phosphomethylpyrimidinekinase	-3,7
S. typhimurium 12468	PMJHGEPJ_03610	purinebindingchemotaxisprotein	-1,1
S. typhimurium 12468	PMJHGEPJ_03248	ecotin	-1,1
S. typhimurium 12468	PMJHGEPJ_00514	outermembraneprotein;outermembranephosphoporinproteinE	-1,0
S. typhimurium 12468	PMJHGEPJ_04389	flagellarhookassociatedprotein2	-1,0
S. typhimurium 12468	PMJHGEPJ_03821	formatedehydrogenase-O,majorsubunit	-0,9
S. typhimurium 12468	PMJHGEPJ_01796	Anaerobicribonucleoside-triphosphatereductase	-0,9
S. typhimurium 12468	PMJHGEPJ_04296	phosphoribosylamine--glycineligase	-0,9

S. typhimurium 12468	PMJHGEPJ_00776	coldshock-likeproteinCspC	-0,9
S. typhimurium 12468	PMJHGEPJ_01139	Nucleoside5-triphosphataseRdgB(dHAPTPdITPXTP-specific)	-0,9
S. typhimurium 12468	PMJHGEPJ_02228	glucose-1-phosphatecytidylyltransferase	-0,8
S. typhimurium 12468	PMJHGEPJ_03151	LppCputativelipoprotein	-0,8
S. typhimurium 12468	PMJHGEPJ_02585	MiaBprotein	-0,8
S. typhimurium 12468	PMJHGEPJ_01754	restrictionenzyme,methylasesubunit	-0,8
S. typhimurium 12468	PMJHGEPJ_00006	23SrRNA(guanine-N-2-)-methyltransferaserlmL	-0,8
S. typhimurium 12468	PMJHGEPJ_02751	NutilizationsubstanceproteinB	-0,8
S. typhimurium 12468	PMJHGEPJ_03058	carbamoylphosphatesynthasesmallsubunit	-0,7
S. typhimurium 12468	PMJHGEPJ_02548	lipoicacidsynthetase	-0,7
S. typhimurium 12468	PMJHGEPJ_03169	Polyribonucleotidenucleotidyltransferase	-0,7
S. typhimurium 12468	PMJHGEPJ_03487	lipopolysaccharidebiosynthesisprotein	-0,7
S. typhimurium 12468	PMJHGEPJ_02230	dTDP-4-dehydrorhamnose3,5-epimerase	-0,7
S. typhimurium 12468	PMJHGEPJ_03353	semialdehydedehydrogenase	-0,7
S. typhimurium 12468	PMJHGEPJ_03745	cytidylatekinase	-0,7
S. typhimurium 12468	PMJHGEPJ_04712	dihydropteroatesynthase2	-0,6
S. typhimurium 12468	PMJHGEPJ_01283	transcriptionaccessoryprotein	-0,6
S. typhimurium 12468	PMJHGEPJ_02853	GMPsynthase	-0,6
S. typhimurium 12468	PMJHGEPJ_00011	3-hydroxydecanoyl-[acyl-carrier-protein]dehydratase	-0,6
S. typhimurium 12468	PMJHGEPJ_03354	erythronate-4-phosphatedehydrogenase	-0,6
S. typhimurium 12468	PMJHGEPJ_04463	polypeptidedeformylase	-0,6
S. typhimurium 12468	PMJHGEPJ_00174	adenylosuccinatelyase	-0,6
S. typhimurium 12468	PMJHGEPJ_00864	recombinaseA	-0,6
S. typhimurium 12468	PMJHGEPJ_03183	ATP-dependentmetalloprotease	-0,6
S. typhimurium 12468	PMJHGEPJ_03218	glutamatesynthasesubunitalpha	-0,6

S. typhimurium 12468	PMJHGEPJ_02524	reductase	-0,5
S. typhimurium 12468	PMJHGEPJ_00278	phenylalanyl-tRNAsynthetasesubunitbeta	-0,5
S. typhimurium 12468	PMJHGEPJ_00661	pseudouridinesynthase	-0,5
S. typhimurium 12468	PMJHGEPJ_03311	NADHdehydrogenaseIchainC;chainD	-0,5
S. typhimurium 12468	PMJHGEPJ_00707	nitrate/nitriteresponseregulatorprotein NarL	-0,5
S. typhimurium 12468	PMJHGEPJ_00376	N-ethylmaleimidereductase	-0,4
S. typhimurium 12468	PMJHGEPJ_03181	PGM/PMMfamilyprotein	-0,4
S. typhimurium 12468	PMJHGEPJ_03172	ribosome-bindingfactorA	-0,4
S. typhimurium 12468	PMJHGEPJ_02391	acriflavinresistanceproteinB	-0,4
S. typhimurium 12468	PMJHGEPJ_03198	putativeABCtransporterauxiliarycomponentYrbC	-0,4
S. typhimurium 12468	PMJHGEPJ_01851	peptidyl-prolylcis-transisomerase	-0,4
S. typhimurium 12468	PMJHGEPJ_01080	thiol:disulfideinterchangeprotein	-0,4
S. typhimurium 12468	PMJHGEPJ_02208	histidinoldehydrogenase	-0,4
S. typhimurium 12468	PMJHGEPJ_01107	phosphoglyceratekinase	-0,4
S. typhimurium 12468	PMJHGEPJ_02739	PreproteintranslocasesubunitYajC	-0,4
S. typhimurium 12468	PMJHGEPJ_02613	phosphoglucomutase	-0,3
S. typhimurium 12468	PMJHGEPJ_04322	transcriptionantiterminationprotein	-0,3
S. typhimurium 12468	PMJHGEPJ_01602	isoleucyl-tRNAsynthetase	-0,3
S. typhimurium 12468	PMJHGEPJ_03435	LD-transpeptidaseYbiS	-0,3
S. typhimurium 12468	PMJHGEPJ_02785	peptidyl-prolylcis-transisomeraseD	-0,3
S. typhimurium 12468	PMJHGEPJ_03671	Glucosamine--fructose-6-phosphateaminotransferase[isomerizing]	-0,3
S. typhimurium 12468	PMJHGEPJ_02782	ATP-dependentclpproteaseATP-bindingsubunitClpX	-0,2
**M consitutitve vs A consitutive**			
**S. typhimurium 12472**			
**Strain**	**Protein ID/FASTA Head**	**Protein Name**	**Expression**
S. typhimurium 12472	HCMLBOHL_04113	potassium-transportingATPasesubunitB	10,9

S. typhimurium 12472	HCMLBOHL_04112	potassium-transportingATPaseAchain	9,4
S. typhimurium 12472	HCMLBOHL_04115	sensorproteinKdpD	8,0
S. typhimurium 12472	HCMLBOHL_01297	ornithinedecarboxylase,inducible	5,9
S. typhimurium 12472	HCMLBOHL_00653	CarboncataboliterepressorCreA	3,4
S. typhimurium 12472	HCMLBOHL_02968	ExportedzincmetalloproteaseYfgCprecursor	2,8
S. typhimurium 12472	HCMLBOHL_02955	GMPsynthase	1,1
S. typhimurium 12472	HCMLBOHL_02954	inosine5'-monophosphatedehydrogenase	1,0
S. typhimurium 12472	HCMLBOHL_02898	phosphoribosylformylglycinamidinesynthase	0,8
S. typhimurium 12472	HCMLBOHL_00274	agmatineureohydrolase	0,8
S. typhimurium 12472	HCMLBOHL_01953	long-chainfattyacidtransportprotein	0,8
S. typhimurium 12472	HCMLBOHL_01139	bifunctionalmethylenetetrahydrofolatedehydrogenase/methenyltetrahydrofolatecyclohydrolase	0,7
S. typhimurium 12472	HCMLBOHL_01407	Dihydroorotase	0,7
S. typhimurium 12472	HCMLBOHL_02850	biotinsynthetase	0,7
S. typhimurium 12472	HCMLBOHL_00774	argininedeiminase	0,7
S. typhimurium 12472	HCMLBOHL_01093	chaperoneproteinHtpG	0,7
S. typhimurium 12472	HCMLBOHL_02939	nucleosidediphosphatekinase(ndk)	0,6
S. typhimurium 12472	HCMLBOHL_02801	hydrolase(HADsuperfamily)	0,6
S. typhimurium 12472	HCMLBOHL_01242	rarelipoproteinB	0,6
S. typhimurium 12472	HCMLBOHL_01476	adenylosuccinatelyase	0,5
S. typhimurium 12472	HCMLBOHL_04022	cytidylatekinase	0,5
S. typhimurium 12472	HCMLBOHL_03613	CDP-diacylglycerolpyrophosphatase	0,5
S. typhimurium 12472	HCMLBOHL_01475	transcriptionalregulatorPhoP,regulatorofvirulencedeterminants	0,5
S. typhimurium 12472	HCMLBOHL_02061	outermembraneprotein	0,5
S. typhimurium 12472	HCMLBOHL_02067	uridine5'-monophosphatekinase	0,5
S. typhimurium 12472	HCMLBOHL_03036	xanthine-guaninephosphoribosyltransferase	0,5
S. typhimurium 12472	HCMLBOHL_03838	recombinationassociatedprotein	0,5

S. typhimurium 12472	HCMLBOHL_00629	DnaJprotein	0,5
S. typhimurium 12472	HCMLBOHL_03137	glycine-tRNAsynthetasesubunitalpha	0,5
S. typhimurium 12472	HCMLBOHL_01059	Lonprotease	0,5
S. typhimurium 12472	HCMLBOHL_04640	DNA-directedRNApolymerasesubunitalpha	0,5
S. typhimurium 12472	HCMLBOHL_02946	GTP-bindingproteinEngA	0,5
S. typhimurium 12472	HCMLBOHL_01537	nitroreductase	0,5
S. typhimurium 12472	HCMLBOHL_03304	ribose-phosphatepyrophosphokinase	0,4
S. typhimurium 12472	HCMLBOHL_04609	NADP-dependentmalatedehydrogenase	0,4
S. typhimurium 12472	HCMLBOHL_01052	cytochromeOubiquinoloxidasesubunitII	0,4
S. typhimurium 12472	HCMLBOHL_04158	FdhEprotein	0,4
S. typhimurium 12472	HCMLBOHL_02433	glucose-1-phosphatecytidylyltransferase	0,4
S. typhimurium 12472	HCMLBOHL_02851	adenosylmethionine--8-amino-7-oxononanoateaminotransferase	0,4
S. typhimurium 12472	HCMLBOHL_01266	MiaBprotein	0,4
S. typhimurium 12472	HCMLBOHL_00153	ADP-heptosesynthase	0,4
S. typhimurium 12472	HCMLBOHL_02609	33kDachaperonin(Heatshockprotein33)(HSP33)	0,4
S. typhimurium 12472	HCMLBOHL_01932	erythronate-4-phosphatedehydrogenase	0,4
S. typhimurium 12472	HCMLBOHL_00873	adenylosuccinatesynthetase	0,4
S. typhimurium 12472	HCMLBOHL_01313	3-hydroxydecanoyl-[acyl-carrier-protein]dehydratase	0,4
S. typhimurium 12472	HCMLBOHL_01701	glutaredoxin	0,4
S. typhimurium 12472	HCMLBOHL_03184	Rhodanese-relatedsulfurtransferase	0,4
S. typhimurium 12472	HCMLBOHL_01889	NADHdehydrogenaseIchainC;chainD	0,4
S. typhimurium 12472	HCMLBOHL_01460	transcription-repaircouplingfactor(TrcF)	0,4
S. typhimurium 12472	HCMLBOHL_02372	alcoholdehydrogenaseclassIII	0,4
S. typhimurium 12472	HCMLBOHL_04043	asparaginyl-tRNAsynthetase	0,4
S. typhimurium 12472	HCMLBOHL_01228	lipoicacidsynthetase	0,4
S. typhimurium 12472	HCMLBOHL_04697	RNApolymerasesigma-Efactor(sigma-24)	0,4
S. typhimurium 12472	HCMLBOHL_01543	exodeoxyribonucleaseIII	0,4

S. typhimurium 12472	HCMLBOHL_04001	thioredoxinreductase	0,4
S. typhimurium 12472	HCMLBOHL_01024	riboflavinbiosynthesisproteinRibD	0,4
S. typhimurium 12472	HCMLBOHL_03604	superoxidedismutase	0,3
S. typhimurium 12472	HCMLBOHL_04040	aspartateaminotransferase	0,3
S. typhimurium 12472	HCMLBOHL_01094	adenylatekinase	0,3
S. typhimurium 12472	HCMLBOHL_02293	Orotidine5'-phosphatedecarboxylase	0,3
S. typhimurium 12472	HCMLBOHL_02964	uracilphosphoribosyltransferase	0,3
S. typhimurium 12472	HCMLBOHL_03803	typeIIIrestriction-modificationsystemStyLTIenzymemod	0,3
S. typhimurium 12472	HCMLBOHL_02301	enoyl-(acylcarrierprotein)reductase	0,3
S. typhimurium 12472	HCMLBOHL_01890	NADHdehydrogenaseIchainB	0,3
S. typhimurium 12472	HCMLBOHL_01316	outermembraneproteinA	0,3
S. typhimurium 12472	HCMLBOHL_01394	glucanbiosynthesisproteinG	0,3
S. typhimurium 12472	HCMLBOHL_02134	pyruvatedehydrogenaseE1component	0,3
S. typhimurium 12472	HCMLBOHL_03364	transcriptionalregulatorKdgR	0,3
S. typhimurium 12472	HCMLBOHL_04020	3-phosphoshikimate1-carboxyvinyltransferase	0,3
S. typhimurium 12472	HCMLBOHL_04705	tRNA/rRNAmethyltransferaseYfiF	0,3
S. typhimurium 12472	HCMLBOHL_00988	tyrosineaminotransferase,tyrosinerepressible	0,3
S. typhimurium 12472	HCMLBOHL_02189	ATP-dependenthelicaseHepA	0,3
S. typhimurium 12472	HCMLBOHL_01232	D-alanyl-D-alaninecarboxypeptidasedacA	0,3
S. typhimurium 12472	HCMLBOHL_02615	transcriptionaccessoryprotein	0,3
S. typhimurium 12472	HCMLBOHL_00830	putativeproteinYtfN	0,3
S. typhimurium 12472	HCMLBOHL_00555	DNA-bindingproteinStpA	0,3
S. typhimurium 12472	HCMLBOHL_04359	malatedehydrogenase	0,3
S. typhimurium 12472	HCMLBOHL_01430	pseudouridylatesynthase	0,3
S. typhimurium 12472	HCMLBOHL_00630	DnaKprotein	0,3
S. typhimurium 12472	HCMLBOHL_02128	bifunctionalaconitatehydratase2/2-methylisocitratedehydratase	0,3
S. typhimurium 12472	HCMLBOHL_04338	biotincarboxylase	0,3

S. typhimurium 12472	HCMLBOHL_03136	glycine-tRNAsynthetasesubunitbeta	0,2
S. typhimurium 12472	HCMLBOHL_01282	glutaminyl-tRNAsynthetase	0,2
S. typhimurium 12472	HCMLBOHL_00766	valyl-tRNAsynthetase	0,2
S. typhimurium 12472	HCMLBOHL_02597	3-dehydroquinatesynthase	0,2
S. typhimurium 12472	HCMLBOHL_04193	DNAgyrasesubunitB	0,2
S. typhimurium 12472	HCMLBOHL_01294	phosphoglucomutase	0,2
S. typhimurium 12472	HCMLBOHL_02806	LD-transpeptidaseYbiS	0,2
S. typhimurium 12472	HCMLBOHL_01439	3-oxoacyl-ACPreductase	0,2
S. typhimurium 12472	HCMLBOHL_04625	50SribosomalproteinL14	0,2
S. typhimurium 12472	HCMLBOHL_02133	dihydrolipoamideacetyltransferase	0,2
S. typhimurium 12472	HCMLBOHL_04607	transketolase	-8,2
S. typhimurium 12472	HCMLBOHL_00560	LysMdomain/BONsuperfamilyprotein	-6,9
S. typhimurium 12472	HCMLBOHL_02482	fructose-bisphosphatealdolaseclassI	-6,8
S. typhimurium 12472	HCMLBOHL_03997	initiationfactorIF-1	-5,9
S. typhimurium 12472	HCMLBOHL_04126	GTPaseactivator	-5,8
S. typhimurium 12472	HCMLBOHL_04612	bacterioferritin	-5,8
S. typhimurium 12472	HCMLBOHL_03161	aldehydedehydrogenaseB	-5,7
S. typhimurium 12472	HCMLBOHL_02269	Manganesecatalase	-5,7
S. typhimurium 12472	HCMLBOHL_01290	Flavodoxin1	-5,5
S. typhimurium 12472	HCMLBOHL_02270	ProteinYciE	-5,4
S. typhimurium 12472	HCMLBOHL_02237	respiratorynitratereductase1subunitalp ha	-5,3
S. typhimurium 12472	HCMLBOHL_01497	ABC- typetransporter,periplasmicsubunit	-5,2
S. typhimurium 12472	HCMLBOHL_01822	nitratereductase	-5,1
S. typhimurium 12472	HCMLBOHL_00897	fumaratereductase,iron-sulfurprotein	-4,9
S. typhimurium 12472	HCMLBOHL_00495	[NiFe]hydrogenasemetallocenterassem blyproteinHypE	-4,7
S. typhimurium 12472	HCMLBOHL_01817	catalaseHPII	-4,7
S. typhimurium 12472	HCMLBOHL_02667	glycerol-3-phosphate- bindingperiplasmicprotein	-4,5
S. typhimurium 12472	HCMLBOHL_04358	argininerepressor	-4,3

S. typhimurium 12472	HCMLBOHL_01648	putativedithiobiotinsynthetase	-4,3
S. typhimurium 12472	HCMLBOHL_00189	2,5-diketo-D-gluconicacidreductaseA	-4,3
S. typhimurium 12472	HCMLBOHL_00498	hydrogenaseisoenzymesformationproteinHypB	-4,2
S. typhimurium 12472	HCMLBOHL_02739	virKprotein	-4,1
S. typhimurium 12472	HCMLBOHL_00991	quinoneoxidoreductase	-4,1
S. typhimurium 12472	HCMLBOHL_02154	celldivisionproteinFtsA	-4,1
S. typhimurium 12472	HCMLBOHL_01363	trprepressorbindingprotein	-4,0
S. typhimurium 12472	HCMLBOHL_00698	proteinyjjA	-4,0
S. typhimurium 12472	HCMLBOHL_04444	nitritereductaselargesubunit	-4,0
S. typhimurium 12472	HCMLBOHL_00681	OsmoticallyinducibleproteinOsmY	-3,9
S. typhimurium 12472	HCMLBOHL_00789	Anaerobicribonucleoside-triphosphatereductase	-3,8
S. typhimurium 12472	HCMLBOHL_01006	maltose/maltodextrintransportATP-bindingprotein	-3,7
S. typhimurium 12472	HCMLBOHL_01471	peptidaseT	-3,6
S. typhimurium 12472	HCMLBOHL_02624	4-alpha-glucanotransferase	-3,5
S. typhimurium 12472	HCMLBOHL_01552	NADsynthetase	-3,5
S. typhimurium 12472	HCMLBOHL_01526	proteinyeaG	-3,0
S. typhimurium 12472	HCMLBOHL_00034	lipopolysaccharidetransportperiplasmicproteinLptA	-3,0
S. typhimurium 12472	HCMLBOHL_04696	anti-RNApolymerasesigmafactorSigE	-2,9
S. typhimurium 12472	HCMLBOHL_00296	monooxygenase	-2,9
S. typhimurium 12472	HCMLBOHL_01896	PhosphoglycolatephosphatasePGPase'	-2,7
S. typhimurium 12472	HCMLBOHL_02406	ssrABactivatedgene	-2,7
S. typhimurium 12472	HCMLBOHL_02625	maltodextrinphosphorylase	-2,1
S. typhimurium 12472	HCMLBOHL_04703	Pyruvateformate-lyase	-2,0
S. typhimurium 12472	HCMLBOHL_04015	formateacetyltransferase1	-1,6
S. typhimurium 12472	HCMLBOHL_03524	ketol-acidreductoisomerase	-1,6
S. typhimurium 12472	HCMLBOHL_02288	aconitatehydratase1	-1,4
S. typhimurium 12472	HCMLBOHL_03582	uridinephosphorylase	-1,3

S. typhimurium 12472	HCMLBOHL_03353	PTSsystemmannose-specifictransportersubunitIIAB	-1,2
S. typhimurium 12472	HCMLBOHL_03185	phosphoglyceromutase	-1,2
S. typhimurium 12472	HCMLBOHL_00912	aspartateammonia-lyase	-1,0
S. typhimurium 12472	HCMLBOHL_04323	maltoseABCtransporterperiplasmicprotein	-0,9
S. typhimurium 12472	HCMLBOHL_03861	pyruvatekinaseA	-0,9
S. typhimurium 12472	HCMLBOHL_00896	fumaratereductaseflavoproteinsubunit	-0,9
S. typhimurium 12472	HCMLBOHL_01021	nucleosidechannelphageT6/colicinKreceptor	-0,8
S. typhimurium 12472	HCMLBOHL_03655	catalase	-0,8
S. typhimurium 12472	HCMLBOHL_03360	coldshock-likeproteinCspC	-0,8
S. typhimurium 12472	HCMLBOHL_03892	methylacceptingchemotaxisproteinII,aspartatesensor-receptor	-0,7
S. typhimurium 12472	HCMLBOHL_00284	fructose1,6-bisphosphatealdolase	-0,7
S. typhimurium 12472	HCMLBOHL_03629	triosephosphateisomerase	-0,6
S. typhimurium 12472	HCMLBOHL_00385	Decarboxylasefamilyprotein	-0,6
S. typhimurium 12472	HCMLBOHL_00675	Deoxyribose-phosphatealdolase	-0,6
S. typhimurium 12472	HCMLBOHL_03131	coldshockprotein	-0,6
S. typhimurium 12472	HCMLBOHL_01809	6-phosphofructokinase2	-0,6
S. typhimurium 12472	HCMLBOHL_02545	PTSsystem,fructose-specificIIBCcomponent	-0,5
S. typhimurium 12472	HCMLBOHL_00073	ATP-dependentRNAhelicaseDeaD	-0,5
S. typhimurium 12472	HCMLBOHL_02547	ptssystem,fructose-specificIIA/FPRcomponent	-0,5
S. typhimurium 12472	HCMLBOHL_01831	outermembraneproteinC	-0,5
S. typhimurium 12472	HCMLBOHL_04131	glutaminesynthetase	-0,5
S. typhimurium 12472	HCMLBOHL_03634	glycerolkinase	-0,4
S. typhimurium 12472	HCMLBOHL_03769	regulatoroflengthofO-antigencomponentoflipopolysaccharide chains	-0,4
S. typhimurium 12472	HCMLBOHL_01657	pyridinenucleotidetranshydrogenasesubunit-beta	-0,3
S. typhimurium 12472	HCMLBOHL_04023	30SribosomalproteinS1	-0,3

S. typhimurium 12472	HCMLBOHL_02654	putativedehydrogenase	-0,3
S. typhimurium 12472	HCMLBOHL_02012	putativedye-decolorizingperoxidase(DyP)YfeX-likesubgroup	-0,3
S. typhimurium 12472	HCMLBOHL_01899	acetatekinase	-0,3
S. typhimurium 12472	HCMLBOHL_02734	ATP-dependentClpproteaseATP-bindingsubunit	-0,3
S. typhimurium 12472	HCMLBOHL_03530	transcriptionterminationfactorRho	-0,3
S. typhimurium 12472	HCMLBOHL_00283	phosphoglyceratekinase	-0,2
